# Rhythm and Music-Based Interventions in Motor Rehabilitation: Current Evidence and Future Perspectives

**DOI:** 10.3389/fnhum.2021.789467

**Published:** 2022-01-17

**Authors:** Thenille Braun Janzen, Yuko Koshimori, Nicole M. Richard, Michael H. Thaut

**Affiliations:** ^1^Center of Mathematics, Computing and Cognition, Universidade Federal do ABC, São Bernardo do Campo, Brazil; ^2^Music and Health Science Research Collaboratory, Faculty of Music, University of Toronto, Toronto, ON, Canada; ^3^Brain Health Imaging Centre, CAMH, Toronto, ON, Canada; ^4^Faculty of Music, Belmont University, Nashville, TN, United States; ^5^Rehabilitation Sciences Institute, University of Toronto, Toronto, ON, Canada

**Keywords:** Rhythmic Auditory Stimulation, music-based interventions, Neurologic Music Therapy (NMT), rehabilitation, movement, gait, upper extremities, auditory-motor entrainment

## Abstract

Research in basic and clinical neuroscience of music conducted over the past decades has begun to uncover music’s high potential as a tool for rehabilitation. Advances in our understanding of how music engages parallel brain networks underpinning sensory and motor processes, arousal, reward, and affective regulation, have laid a sound neuroscientific foundation for the development of theory-driven music interventions that have been systematically tested in clinical settings. Of particular significance in the context of motor rehabilitation is the notion that musical rhythms can entrain movement patterns in patients with movement-related disorders, serving as a continuous time reference that can help regulate movement timing and pace. To date, a significant number of clinical and experimental studies have tested the application of rhythm- and music-based interventions to improve motor functions following central nervous injury and/or degeneration. The goal of this review is to appraise the current state of knowledge on the effectiveness of music and rhythm to modulate movement spatiotemporal patterns and restore motor function. By organizing and providing a critical appraisal of a large body of research, we hope to provide a revised framework for future research on the effectiveness of rhythm- and music-based interventions to restore and (re)train motor function.

## Introduction

Brain and clinical research conducted over the past 25 years have provided a new understanding of the capabilities of music to engage and shape non-musical perceptual, cognitive, language, and motor functions to effectively support brain recovery processes (Thaut, 2010; Koshimori and Thaut, 2018, 2019; Altenmüller and James, 2020; Thaut and Koshimori, 2020; Chatterjee et al., 2021). In the context of motor rehabilitation, the finding that musical rhythm entrains movement in patients with neurological disorders opened new frontiers for the use of rhythm and music as a continuous time reference to prime the motor system and re-program the execution of movement patterns (Thaut et al., 1996, 2015).

Music is a potent driving force for movement. Synchronization of body movements to external rhythmic auditory stimuli, such as music or a metronome, is possible because the regular and predictable rhythmic structure of the music is readily and precisely detected by the auditory system, inducing entrainment of neuronal activity in auditory and motor regions of the brain involved in rhythm perception and movement production (Thaut et al., 2015; Damm et al., 2020). Growing experimental evidence of the effect of rhythmic entrainment on movement spatiotemporal patterns and the current advances of the neural underpinnings of auditory-motor coupling have informed the development of theory-driven interventions that have been tested in a large number of studies.

In this paper, we review recent studies focusing on four evidence-based interventions using rhythm and active music playing to improve motor functions following central nervous injury and/or degeneration: respectively, Rhythmic Auditory Stimulation, and Music-supported Therapy, Therapeutic Instrumental Music Performance, and Patterned Sensory Enhancement (Table 1). This paper aims to provide a critical narrative review of the current literature on the effects of rhythm- and music-based interventions for motor rehabilitation in a wide range of clinical populations (e.g., Parkinson’s Disease, stroke, cerebral palsy, traumatic brain injury, and multiple sclerosis) as well as aging. Additionally, considering that evidenced-based practices are built on ongoing fundamental brain research and theories, we briefly overview recent neurophysiological and neuroimaging evidence of the potential mechanisms underlying the effectiveness of rhythm and music in shaping movement timing and control. To finalize, we highlight research questions and methodological concerns that should be on the agenda for future research. By organizing and providing a critical appraisal of a large body of research, we hope to provide a revised framework for future research on the effectiveness of rhythm- and music-based interventions to restore and (re)train motor function.

**TABLE 1:** Description of interventions.

Intervention	Description
*Rhythmic Auditory Stimulation (RAS)* (Thaut et al., 1996)	RAS is a Neurologic Music Therapy rhythm-based rehabilitation technique designed to facilitate the rehabilitation of intrinsically rhythmic movements through rhythmic auditory cues, such as metronome beats or music with embedded metronome. The rhythmic cues are first matched to each patient’s preferred gait cadence and gradually increased/decreased 5–10% to encourage rhythmic entrainment.
*Music-supported Therapy (MST)* (Schneider et al., 2007)	MST is based on music playing as a rehabilitation tool to train fine and gross movement of the paretic upper extremity. Training sessions consist of playing an electronic keyboard and/or drum pads where exercises involve melodic sequences that vary in the number of tones, movement velocity, and type of movement. Exercises progressively increase in difficulty until patients learn to play songs.
*Therapeutic Instrumental Music Performance (TIMP)* (Thaut and Hoemberg, 2014)	TIMP is a Neurologic Music Therapy technique that involves playing musical instruments to exercise and stimulate functional movement patterns. In this technique, musical instruments such as drums or keyboard are not played in a traditional manner but are rather placed in strategic locations relative to the patient’s body to train range of motion, endurance, strength, functional hand movements, finger dexterity, and limb coordination. Training exercises involve a strong rhythmic component whereby metronome or music are used to provide rhythmic cues to facilitate auditory-motor entrainment. Cueing frequency is initially matched to the patient’s comfort level and gradually decreased/increased depending on the therapy goal.
*Patterned Sensory Enhancement (PSE)* (Thaut and Hoemberg, 2014)	PSE is a Neurologic Music Therapy technique that takes advantage of the rhythmic, melodic, harmonic, and dynamic-acoustical elements of music to provide temporal, spatial, and force cues. This technique uses musical patterns to structure and regulate movement patterns and can be applied to movements that are not rhythmical by nature (e.g., arm and hand movements, functional movement sequences such as dressing or sit-to-stand transfers).

## Clinical Applications

### Rhythmic Auditory Stimulation

The presentation of rhythmic auditory cues as means to facilitate movement and promote sustained functional changes in patients with motor impairment has been extensively investigated (reviewed in Sihvonen et al., 2017; Ghai et al., 2018a; Ghai and Ghai, 2019; Schaffert et al., 2019). Rhythmic Auditory Stimulation (RAS) is a Neurologic Music Therapy (NMT) rehabilitation technique that involves the presentation of auditory rhythmic cues in the form of repetitive isochronous pulses (e.g., metronome clicks) or metrically accentuated music with an embedded metronome to promote auditory-motor entrainment of intrinsically rhythmic movements (Thaut and Hoemberg, 2014; Thaut et al., 2015). Typically, rhythmic cues are matched to the individual’s preferred cadence and, once the movement is entrained to the external cues, the rhythm is gradually increased or decreased by 5–10% over baseline (Thaut et al., 1996; Nombela et al., 2013).

A series of seminal research studies conducted in the 1990s demonstrated that auditory rhythms prime the motor system by providing anticipatory time cues that allow movement planning and preparation, thus helping to regulate walking timing and pace in healthy older adults as well as in individuals with Parkinson’s Disease (Thaut et al., 1996; McIntosh et al., 1997), stroke (Thaut et al., 1993, 1997; Prassas et al., 1997), traumatic brain injury (Hurt et al., 1998) and cerebral palsy (Thaut et al., 1998). These findings have been replicated in an increasing number of studies, building a robust body of experimental and clinical evidence on the application of RAS as a rehabilitation tool for gait disorders in Parkinson’s Disease (Ghai et al., 2018a), stroke (Ghai and Ghai, 2019), traumatic brain and spinal cord injury (Magee et al., 2017), multiple sclerosis (Ghai and Ghai, 2018), cerebral palsy (Ghai et al., 2018b), and older adults (Ghai et al., 2018c). As the scope of this paper does not allow for a thorough description of all clinical research published to date – many of which have already been examined in past systematic reviews and meta-analyses – in the following sections we highlight findings that have been consistently reported across studies and focus the review on articles published in English in the past 5 years (Supplementary Table 1). Our goal is to underline points of consensus in the literature and call attention to areas of research that are yet to be fully explored (Table 2).

**TABLE 2:** Current state of knowledge: summary of current findings on the therapeutic effects of rhythm- and music-based interventions (RAS, MST, TIMP, and PSE) on motor rehabilitation.

	Main Findings
*Rhythmic Auditory Stimulation (RAS)*	° There is consistent evidence supporting the use of RAS for gait training in PD and sub-acute stroke, with repeated reports of significant improvements in gait spatiotemporal parameters (velocity, cadence, stride length). ° Recent studies with PD patients suggest positive effects on balance, freezing of gait, overall motor functioning, fear of fall, and number of falls. ° Emerging findings indicate that RAS optimizes conventional therapies, such as Deep Brain Stimulation, treadmill training, and motor imagery. ° However, there is a paucity of randomized controlled studies on the effectiveness of RAS for the treatment of gait impairments in acute stages of stroke, TBI, MS, CP, dementia or Alzheimer’s Disease, and older adults. Limited research is available on the effectiveness of RAS for the treatment of gait impairment in children.
*Music-supported Therapy (MST)*	° There is a strong body of research on the effectiveness of MST to improve functional movements of the paretic upper extremity in sub-acute and chronic stroke. However, there are no clinical studies in other populations. ° Further research is needed to better determine optimal MST treatment intensity and duration depending on the stroke stage. ° In relation to the application of music practice (e.g., piano/keyboard lessons, drumming), there is currently little research evidence on the effectiveness of active music playing to improve motor function in CP, TBI, MS, and older adults.
*Therapeutic Instrumental Music Performance (TIMP)*	° There is emerging evidence on the benefits of TIMP for upper extremity rehabilitation in stroke, with preliminary results suggesting significant improvements in fine and gross motor function. ° Recent studies indicate the feasibility of TIMP-based interventions for CP and PD; however, little is yet known about the potential application in other populations.
*Patterned Sensory Enhancement (PSE)*	° There is growing evidence of the benefits of rhythmically cued PSE exercises to improve upper extremity function in stroke, with recent findings showing associations between improved function and better regulation of muscle activation patterns of the paretic limb. ° Initial findings also demonstrate the effectiveness of PSE training for gross motor capacity for sit-to-stand movements in CP. No studies are yet available on other clinical populations.

#### Parkinson’s Disease

Parkinson’s Disease (PD) is characterized primarily by a dysfunctional basal ganglia system that results in motor impairments including rigidity, bradykinesia, and/or resting tremor in the early disease stage. In advanced PD, recurrent falls, postural impairment, unstable balance, and freezing of gait (FOG) are often observed in addition to gait dysfunctions such as reduced gait velocity and stride length, increased cadence, and irregular timing of walking pace (Morris et al., 1994, 2001; Luquin et al., 2017). Typically, the primary treatment for motor symptoms in PD includes pharmacological interventions, such as dopamine replacement therapy (Oakes et al., 2004; Fasano et al., 2012). However, motor symptoms observed in more advanced cases tend to have limited responses to conventional therapies (Luquin et al., 2017).

Recent meta-analyses and systematic reviews agree that RAS is an effective tool to improve spatiotemporal gait parameters, enhancing gait velocity and stride length, and reducing gait cadence (reviewed in Spaulding et al., 2013; Rocha et al., 2014; Ghai et al., 2018a; Zhou et al., 2021). Studies repeatedly show that, in the presence of auditory rhythmic cues, PD patients typically walk faster and with increased step length. According to Ghai et al. (2018a), a clinical dosage of three to five 20–40 min-sessions per week is most effective for this population.

More recently, there is growing evidence that RAS training is effective to improve motor function such as balance, FOG, motor performance, and recurrence of falls. A recent randomized control trial (RCT) included 154 participants with early to mid-stage PD (H&Y 1–3) who were assigned to three different interventions: multimodal balance training with RAS and without RAS, and an educational program as a control intervention (Capato et al., 2020a). The training consisted of two 45-min weekly sessions over 5 weeks. RAS was presented using a metronome with varying speeds depending on the type of exercise. Results indicated that both active interventions provided significant improvement on balance at post-training compared to baseline and to control. However, balance improvement was greater for the RAS group compared to the group that received training without rhythmic cues. Another notable benefit of the RAS-assisted balance training was that only the RAS group showed a carry-over effect at a 6-month follow-up compared to the baseline measurement. This same multimodal balance training with and without RAS was implemented with more severe cases of PD (H&Y 4) (Capato et al., 2020b). Similarly, the results indicated positive effects from both active interventions in improving balance, however, the beneficial effects were maintained only in the RAS group at the 6-month follow-up.

Rhythmic auditory cueing has been shown to effectively improve FOG (Capato et al., 2020a; Horin et al., 2020; Naro et al., 2020; for a review of earlier studies, see Ginis et al., 2018). Capato et al. (2020a) reported that the benefits of treadmill training with RAS were observed in PD patients with and without FOG. It has also been demonstrated that RAS-assisted interventions reduce the scores of MD-UPDRS III (Bailey et al., 2018; Calabrò et al., 2019; Naro et al., 2020). Importantly, the benefits of RAS training on the MD-UPDRS III were retained at a 6-month follow-up (Capato et al., 2020a) and at a 1-month follow-up in more severe PD (Capato et al., 2020b), results which were not observed in the active control groups without RAS. Interestingly, emerging evidence also suggests that RAS training complements the effects of conventional therapies for PD, including deep brain stimulation, by significantly enhancing gait velocity and stability when these interventions are combined (Gooßes et al., 2020; Naro et al., 2020).

Recent evidence also suggests that RAS-assisted gait training significantly reduces patients’ overall fear of falling (Thaut et al., 2019; Capato et al., 2020b; Naro et al., 2020; Cochen De Cock et al., 2021) and the number of falls (Thaut et al., 2019). Using a randomized withdrawal/discontinuation design, a recent study investigated the effect of RAS on incidences of falls in 47 participants with moderate to severe stages of PD (H&Y 3 and 4) with a history of falls (Thaut et al., 2019). One group (*n* = 25) trained daily with RAS for 24 weeks, while the other group (*n* = 22) undertook the same training but discontinued the protocol between weeks 8 and 16 and then resumed the training for the last 8 weeks of the intervention. During the training, participants walked for 30 min in a home-based environment with a metronome click embedded in folk and classical instrumental music with a strong 2/4 tempo. The first 8 weeks of training significantly reduced the number of falls in both groups. Discontinuation of the RAS training between weeks 8 and 16 resulted in a significant increase in incidences of falls in the control group. Moreover, once RAS training resumed, the number of falls decreased again in the control group.

The effects of auditory cueing on stride length variability, on the other hand, have shown inconsistent results. Some studies report improvements (Dalla Bella et al., 2017; Chang et al., 2019; Erra et al., 2019; Park et al., 2021) while others report that external auditory rhythm increases stride variability (Harrison et al., 2019; Lirani-Silva et al., 2019; Horin et al., 2020). This inconsistency in the results may be partly due to the selected rhythm cadence. Studies reporting beneficial effects typically employ individualized optimal tempi based on participant’s preferred cadence (e.g., ±10% of preferred cadence), whereas studies reporting negative effects on stride length variability used tempi of preferred cadence (Harrison et al., 2019; Lirani-Silva et al., 2019; Horin et al., 2020). These findings indicate that it is important to optimize RAS cadence individually to investigate the effects of external auditory cueing on stride length variability. These mixed results may also be associated with significant baseline differences in stride length variability among study participants and with patients’ rhythmic abilities and musical training (Dalla Bella et al., 2017; Cochen De Cock et al., 2018). Further research on this topic is warranted.

#### Stroke

Impairments in motor function such as decreased postural stability, gait dysfunctions, and impaired upper-limb function are common consequences of stroke (Langhorne et al., 2009). Over the years, a considerable body of literature has investigated the effectiveness of RAS for lower limb rehabilitation in stroke (reviewed in Nascimento et al., 2015; Magee et al., 2017; Ghai and Ghai, 2019; le Perf et al., 2019). Clinical research on sub-acute and chronic stroke patients have consistently shown beneficial effects of RAS training on gait spatiotemporal parameters, with significant improvements in gait velocity, stride length, cadence, and postural stability (Nascimento et al., 2015; Magee et al., 2017; Ghai and Ghai, 2019). Overall, it has been suggested that maximum benefits are observed with training protocols consisting of 20- to 40-min sessions repeated three to five times a week (Ghai and Ghai, 2019).

Findings from recent RCTs suggest that RAS training also optimizes the effects of other therapeutic strategies for post-stroke gait (Lee et al., 2018; Mainka et al., 2018; Wang et al., 2021). For instance, Mainka et al. (2018) randomly allocated 35 stroke patients to three groups: treadmill training with or without RAS and therapy based on the Bobath approach. All groups received 4 weeks of intervention in addition to conventional therapy. Rhythmic cues consisted of metronome-embedded music adjusted to each patients’ cadence. Post-treatment assessments revealed that gait velocity and cadence improved more in patients in the RAS training compared to the other therapeutic groups. This finding adds to existing evidence of the beneficial effects of combining RAS with other adjunct therapeutic strategies for gait (for further discussion, see Ghai and Ghai, 2019).

The vast majority of studies addressing the effectiveness of RAS on gait focuses on the sub-acute and chronic stages of stroke (Ghai and Ghai, 2019, for review of earlier studies). The first 2 weeks post-stroke is generally defined as the acute stage of stroke, whereas the sub-acute stage refers to 3–11 weeks post-stroke, the early chronic stage comprises 12–24 weeks post-stroke and more than 24 weeks after the stroke is classified as the chronic stage of stroke (Rehme et al., 2012). Recently, Gonzalez-Hoelling et al. (2021) examined the effects of RAS gait training in patients after 4–21 days from stroke onset. Of the 55 participants, 28 in-patients were assigned to a rehabilitation program consisting of conventional therapy combined with RAS gait training conducted for 90 min, three times a week. The duration of the intervention varied according to the patient’s hospitalization duration, with participants completing between three and 34 sessions (average of 14 sessions). The study indicated that, at discharge, all patients improved significantly in measures of gait, balance, and walking ability, with no significant differences between conventional therapy combined with RAS training. The authors noted, however, that patients in the combined-RAS training showed more improvement in functional ambulation, walking ability, and independent walking than participants in the conventional rehabilitation program. Nevertheless, Gonzalez-Hoelling and colleagues reported that all patients in the RAS-training group were unable to walk at the baseline assessment and that some patients did not tolerate the additional therapy sessions required for the RAS training, which may have limited the gains in the RAS group. In previous RAS studies with individuals in early post-stroke (Thaut et al., 1997, 2007), only patients who were able to complete five stride cycles with handheld assistance were enrolled in the program. For instance, in Thaut et al. (2007), 43 patients within 21 days of stroke onset received RAS gait training for 30 min, five times a week, for 3 weeks, while another group of acute stroke patients (*n* = 35) received an active control therapy based on Bobath therapy for the same duration. RAS training consisted of music with an embedded metronome matched to each patient’s baseline cadence, with rhythm frequency increased by 5%. Study findings demonstrated higher gains for patients in the RAS training than the control group in measures of gait velocity, stride length, cadence, and gait symmetry after 3 weeks of intervention (Thaut et al., 2007). Taken together, these studies suggest that patients’ motor function at enrollment need to be carefully considered for an appropriate adherence to the protocol, ensuring that the duration of the training, task difficulty, and cueing frequency is adjusted according to each individual’s cognitive and motor capacity as well as the clinical stage. Moreover, it has also been shown that patients’ rhythm abilities may significantly interact with the strength of the response to rhythmic cueing (Crosby et al., 2020). Further research is warranted to better determine the earliest stage at which RAS training would be feasible and effective in stroke.

#### Other Clinical Applications

The effect of auditory-motor entrainment on gait performance in PD and stroke is well-documented and the clinical evidence of its effectiveness for gait training is robust. In the past years, there has been growing interest in the application of RAS as a motor rehabilitation technique in other neurological conditions where gait and postural stability are affected, including traumatic brain injury, multiple sclerosis, cerebral palsy, Alzheimer’s disease, as well as in aging (Thaut and Abiru, 2010; Magee et al., 2017; Sihvonen et al., 2017).

Impairments in gait are common after traumatic brain injury (TBI) resulting in reduced walking speed, stride length, and cadence, as well as increased step-to-step variability and abnormal muscle activation patterns of lower extremity muscles (Williams et al., 2010; Acuña et al., 2018; Galea et al., 2018). To date, few clinical studies have examined the feasibility and effectiveness of RAS training for gait rehabilitation in this population (Hurt et al., 1998; Goldshtrom et al., 2010; Kim et al., 2016; Sheridan et al., 2021; Thompson et al., 2021). In the first study to examine the effects of RAS training in TBI (Hurt et al., 1998), a 5-week home RAS gait training was provided to five community-dwelling adults with post-TBI (experiment 2). All patients in the study were able to walk independently or with an assistive device without physical assistance from the therapist. RAS consisted of metronome pulses embedded in rhythmically accented music set at 5% over each patient’s fast walk cadence. Post-test assessments indicated a significant improvement in speed, stride length, and cadence of preferred pace gait after 5 weeks of RAS training (Hurt et al., 1998). Recent case reports and feasibility studies have further examined the potential of RAS training in this population (Sheridan et al., 2021; Thompson et al., 2021). For instance, in Thompson et al. (2021), 10 individuals 1–20 years post-TBI were enrolled in a RAS gait program consisting of daily 30-min training for 2 weeks, totaling 10 treatment sessions. The study pointed to the feasibility of RAS for gait training with those individuals who were able to ambulate for 30 min without assistive devices and reported positive trends toward changes in gait parameters such as velocity, step length, cadence, and 10-min walk speed, with sustained improvements at a 1-week follow-up assessment. More research is needed to build a stronger body of clinical evidence of the potential applications of RAS in TBI gait rehabilitation.

There is increasing research on the potential for RAS to help address gait and postural dysfunctions in multiple sclerosis (MS) (reviewed in Ghai and Ghai, 2018; Vinciguerra et al., 2019; Lopes and Keppers, 2021). MS is a chronic demyelinating disease of the central nervous system affecting sensory, motor, and cognitive functioning (Benedetti et al., 1999; Comber et al., 2017; Oreja-Guevara et al., 2019). Recent studies have shown that persons with MS with mild to moderate motor impairments are able to synchronize their steps to music or a metronome at a range of different tempi (Moumdjian et al., 2019a,b), however, higher gait synchronization is found when the auditory rhythm is set at +8 and +10% of preferred cadence (Moumdjian et al., 2020). Clinical studies have successfully implemented RAS in gait rehabilitation in this clinical population. In Shahraki et al. (2017), 18 patients were randomly allocated to two groups: one group performed gait training with RAS at +10% of preferred cadence for 3 weeks, with 30-min sessions three times a week, while the control group performed similar gait exercises without RAS. The results of the study suggested significant differences between groups, with higher gains in stride length, stride time, cadence, and gait speed in RAS training compared to conventional gait intervention. RAS training also has been shown to complement the effects of other therapeutic strategies, such as motor imagery (Seebacher et al., 2017, 2019) and treadmill training (Maggio et al., 2021). Motor imagery combined with rhythmic cueing was investigated in a large RCT with 112 persons with MS (Seebacher et al., 2017). Participants in the RAS-combined home-based training were asked to kinesthetically imagine the execution of a movement with music or metronome-induced rhythmic auditory cueing for 17 min, six times a week, for a total of 4 weeks. Findings indicated that music- and metronome-cued motor imagery significantly improved gait spatiotemporal parameters compared to control, with positive effects on walking speed and the 6-min walking test, suggesting reduced physical fatigue. Further, results also showed significant improvements in quality of life, pain, physical and mental health in persons with MS after metronome/music-cued motor imagery intervention compared to the control intervention (Seebacher et al., 2017). In a subsequent study, Seebacher et al. (2019) reported that providing additional verbal cues increases the effectiveness of music-cued mental imagery training for individuals with mild to moderate MS. The beneficial effects of gait therapy combined with RAS have also been shown (Maggio et al., 2021). Ten persons with MS were assigned to an intervention consisting of 30 min of treadmill training with RAS conducted three times per week for a total of 8 weeks, while 10 patients received conventional overground gait training for the same amount of time. Post-intervention assessments indicated significant changes from baseline in measures of static and dynamic balance, gait velocity, and mobility for the RAS-combined intervention. Furthermore, results pointed to significant improvements in non-motor aspects, including mood, perception of quality of life, as well as physical and mental health for patients in the RAS training group (Maggio et al., 2021).

To date, there is limited research on the effects of RAS gait training for individuals with cerebral palsy (CP) (Ghai et al., 2018b, for further review). CP is a developmental disorder characterized by pre/postnatal brain damage and is considered the most common cause of physical disability in childhood, often affecting gait function including shorter step length, increased step variability, poor dynamic gait stability, and slower gait velocity compared to typically developing children at the same age (Katz-Leurer et al., 2009; Pakula et al., 2009; Kurz et al., 2012). Recently, an experimental study investigating the association between rhythm perception and gait characteristics in children with CP and typically developing children reported no significant differences in rhythm perception abilities between groups, also finding that children in both groups successfully synchronized their steps to a metronome 7.5% faster or slower than preferred cadence (Schweizer et al., 2020). Initial research findings indeed reported positive effects of RAS on gait training for children and adolescents with CP (Thaut et al., 1998; Kwak, 2007; Baram and Lenger, 2012). However, no clinical studies on the effects of RAS have been conducted in this pediatric population in the past 5 years, revealing an area yet to be fully explored.

Rhythmic Auditory Stimulation gait training has been examined to some extent in adults with spastic CP (Kim et al., 2011, 2012, 2020; Varsamis et al., 2012; Efraimidou et al., 2016), where deficits in independent walking, bilateral control, as well as pain and fatigue are often associated with a decline in mobility in the adult population. In a series of clinical studies, Kim and colleagues reported that a 3-week RAS training improved functional gait in measures such as cadence, stride length, and gait velocity, also promoting significant kinematic changes of the pelvic and hip movement (Kim et al., 2011, 2012). More recently, Kim et al. (2020) examined whether the musical properties of the rhythmic cues would influence the effectiveness of the training. A total of 13 young adults with diplegic CP received 30 min of RAS training, three times per week for 4 weeks. The rhythmic cues for one group consisted of simple chord progressions emphasizing the metronome tempo, whereas the cues presented to the second group included more complex chord progressions and a melodic structure along with the metronome. Analysis of spatiotemporal and kinematic gait parameters revealed significant improvements in both groups in measures of cadence, velocity, stride length, as well as changes in the minimal flexion angle of the hip joint and increased hip extension at terminal stance. These findings thus corroborate the evidence that the predictable rhythmic structure of the auditory cues is the primary driving agent of change in gait parameters. Nevertheless, the study reported group differences in the range of the ankle motion, with more improvement in maximal ankle plantar flexion in the pre-swing phase in the complex chord group compared to simple chord cues. The authors suggested that musically complex cues may facilitate engagement and enhance the drive to move. Indeed, Thaut et al. (1997) proposed that the musical texture of the cues provide additional timing information that may facilitate detection, anticipation, and synchronization to the rhythmic cues. Further research with biomechanical measures would be of interest to better understand how specific parameters of the acoustic cues can facilitate auditory-motor entrainment.

Gait disturbances, postural instability as well as increased risk of falls are frequently observed in mild to moderate dementia and Alzheimer’s disease (AD) as a function of the severity of cognitive impairments (IJmker and Lamoth, 2012; Allali et al., 2016; Castrillo et al., 2016; Kikkert et al., 2016). Wittwer et al. (2013) examined the immediate effects of RAS on gait in a study involving 30 older adults with mild to moderately severe AD who were able to ambulate for 100 m on a level surface without a gait aid. Participants were required to walk over an electronic walkway and synchronize their steps either to music or a metronome matched to each individual’s baseline cadence. Assessments of gait spatiotemporal parameters under each condition revealed deleterious effects on gait with a significant decrease in gait velocity and greater stride length variability when walking in time with both types of auditory cues compared to baseline. As discussed earlier, significantly increased gait variability at baseline and the chosen cueing frequency may influence the response to RAS gait training. The authors also raised the possibility that people with dementia may require more practice and longer intervention periods to produce positive benefits on gait parameters (see also Clair and O’Konski, 2006). In a subsequent intervention study, Wittwer et al. (2020) examined the feasibility of a home-based RAS gait training to address movement-related deficits in early AD. Eleven community-dwelling older adults living with AD and able to ambulate were enrolled in an intervention consisting of eight 45-min gait training sessions delivered at home over 4 weeks. Rhythmic cues involved music with a clear temporal structure and progressively modified tempo (±10% preferred cadence). Post-intervention assessments indicated a significant increase in gait velocity associated with improved stride length after the intervention. Moreover, findings also demonstrated that the home-based gait training was associated with high levels of safety, compliance, adherence, and satisfaction, opening the possibility for further investigation on the effects of RAS gait training for people with AD.

Rhythmic cueing has also been investigated in healthy older adults (reviewed in Ghai et al., 2018c; Thaut and Koshimori, 2020). According to a recent meta-analysis and systematic review, RAS significantly enhances spatiotemporal gait parameters such as gait velocity, cadence, and stride length in older adults (≥60 years old) (Ghai et al., 2018c). However, most studies to date only concern the immediate effects of rhythmic auditory entrainment on gait. The direct effect of rhythmic cueing was the primary focus in recent studies showing positive effects on gait variability (Vitorio et al., 2018) and dynamical postural stability in healthy older adults (Minino et al., 2021). Only one clinical RCT has been conducted to examine the effects of RAS gait training for community-dwelling older adults (Trombetti et al., 2011). In this study, 134 adults were randomly allocated to a music-based intervention or a delayed intervention control group. The intervention consisted of 1-h weekly sessions conducted over a 6-month period whereby participants performed a variety of multitasking exercises, such as walking in synchrony with piano music with changes in rhythmic structure. Assessments conducted after 6 months of training revealed improvements in gait performance under single- and dual-task conditions, enhanced balance, and a significant reduction in the number of falls and the risk of falling. Moreover, positive benefits persisted after 6 months of intervention (Trombetti et al., 2011). Considering that decreased gait function and balance in aging are important predictors of falls, more research is needed to better understand the effectiveness of RAS intervention on aging gait.

### Music-Based Interventions

Music-based interventions have emerged as a promising therapeutic approach for the restoration of upper extremity functional abilities in several neurologic conditions. In the past years, an increasing number of clinical studies have assessed the potential rehabilitative effects of music-based interventions involving active music playing to address fine and gross upper extremity motor deficits, with particular focus on stroke (Zhang et al., 2016; Grau-Sánchez et al., 2020, for review) and cerebral palsy (Alves-Pinto et al., 2016). In the following sections, we review clinical evidence published in the past 5 years on the effects of three music-based interventions for the rehabilitation of discrete and non-rhythmic movements, namely Music-supported Therapy (Schneider et al., 2007), Therapeutic Instrumental Music Performance (TIMP) (Thaut, 2005; Thaut and Hoemberg, 2014), and Patterned Sensory Enhancement (PSE) (Thaut et al., 1991; Thaut and Hoemberg, 2014; Table 1). We begin by examining recent literature on stroke and cerebral palsy, which have been at the forefront of research on this topic, followed by an overview of current findings concerning other clinical populations, including Parkinson’s Disease, traumatic brain injury, multiple sclerosis, and aging (Supplementary Table 2).

#### Stroke

Motor deficits of the upper extremity are common in patients with stroke and have a relevant impact on patients’ activities of daily living, independence, and quality of life (Morris et al., 2013). Motor function recovery for this population relies primarily on motor rehabilitation (Langhorne et al., 2011), thus music-based therapies have received ample research attention in the past years (for discussion, see Thaut and McIntosh, 2014; Zhang et al., 2016; Moumdjian et al., 2017; Chen, 2018; Altenmüller and James, 2020; Grau-Sánchez et al., 2020; Huang et al., 2021).

One of the most investigated music-based interventions to treat upper limb hemiparesis after stroke is Music-supported Therapy (MST) (Schneider et al., 2007; Rodriguez-Fornells et al., 2012; reviewed in Grau-Sánchez et al., 2020). This intervention involves playing a keyboard and/or electronic drum using motor sequences of increasing difficulty to train fine and gross motor skills, respectively. The therapeutic technique is based on the premise that playing musical instruments is an enjoyable activity involving complex and coordinated movements that require auditory-motor coupling and integration through real-time multisensory information (Schneider et al., 2007; Rodriguez-Fornells et al., 2012; Ripollés et al., 2016). Initial clinical research suggested that a rehabilitation regimen consisting of fifteen 30-min sessions of active music playing completed over a period of 3 to 4 weeks, in addition to conventional therapy, is effective to improve upper extremity movement parameters such as speed, precision, and smoothness (Schneider et al., 2007, 2010; Altenmüller et al., 2009). These results have been replicated in a growing body of research showing that this intervention protocol significantly improves functional movements of the paretic upper extremity in subacute as well as in chronic stroke (Chong et al., 2017; Grau-Sánchez et al., 2017, 2018; Fujioka et al., 2018; Ghai et al., 2021; for review of earlier studies, see Grau-Sánchez et al., 2020).

Recently, studies examining the progression and retention of motor gains with music-supported therapy have shown that movement velocity, accuracy, and force are rapidly improved within the first training sessions involving simple movement sequences on the piano (Grau-Sánchez et al., 2017). However, functional gains and transfer to everyday tasks are observed after at least 4 weeks of intervention and are even more noticeable after a second training period, thus suggesting progressive improvements (Grau-Sánchez et al., 2018, 2020). Importantly, recent intervention protocols have indicated that treatment intensity and duration may need to be increased to promote effective motor recovery for patients in the chronic stage of stroke (Grau-Sánchez et al., 2021).

When comparing the effectiveness of music-supported therapy with conventional therapy, a recent RCT has shown that active music playing promotes the same retention of gains as standard rehabilitation programs when the amount of extra therapy received by the control group is the same as the intervention group. Specifically, Grau-Sánchez et al. (2018) randomly assigned 40 subacute stroke patients to two treatment groups: one group received 30-min sessions of music-supported therapy five times per week in addition to their standard rehabilitation program, while the control group received the same amount of additional conventional therapy. Assessments conducted after 4 weeks of intervention and at a 3-month follow-up indicated that both training protocols promoted improvements in motor function and no significant differences between treatment groups were observed at follow-up. Thus, these findings indicate that music-supported therapy is as effective as standard rehabilitation therapy to enhance the motor function and movement kinematics of the paretic upper extremity in the subacute stage of stroke.

There is also increasing research on the potential application of TIMP for upper limb rehabilitation in stroke. TIMP is an NMT technique for sensorimotor training that involves active music playing as a means to train gross or fine motor skills (Thaut and Hoemberg, 2014). Unlike Music-supported Therapy, in this protocol, musical instruments are not played in a conventional way but are rather used as a source of visual, tactile, and auditory feedback as they are positioned in strategic locations relative to the patient’s body to train therapeutic meaningful movements that are transferable to real-world applications, such as trunk rotation, shoulder flexion/extension, elbow flexion/extension, forearm supination/pronation, hand grasp and release, finger individuation, and reaching movements. This intervention has been investigated in recent feasibility and randomized pilot studies with subacute and chronic stroke patients (Raghavan et al., 2016; Street et al., 2018, 2019, 2020; Haire et al., 2021a). In Raghavan et al. (2016), 13 chronic stroke patients completed 45-min group music-based therapy, twice a week, over 6 weeks. The intervention was led by a music therapist and an occupational therapist and included a range of therapeutic meaningful exercises using percussion instruments, piano, and drums to train gross and fine motor skills of the paretic upper extremity. Assessments conducted post-intervention indicated significant improvements from baseline in motor impairment (Fugl-Meyer Test), sensory deficits (Two-point Discrimination test), disability (Modified Ranking Scale), and overall well-being. Importantly, a follow-up assessment suggested retention of gains after 1 year of treatment completion (Raghavan et al., 2016). Haire et al. (2021a) recently examined the effects of a TIMP-based intervention consisting of nine sessions for 30 community-dwelling individuals with sustained unilateral stroke. The study included three groups where active TIMP exercises were combined with mental imagery with and without metronome cueing. The findings indicated significant gains in paretic arm control after 3 weeks of intervention as measured by the Fugl-Meyer and Wolf Motor Function tests.

The feasibility of a TIMP home-based intervention was examined in a study with 10 subacute stroke patients (Street et al., 2018). Analysis of structured interviews indicated that a 6-week intervention for arm rehabilitation was considered highly motivating, with high tolerance and adherence to treatment, as well as non-fatiguing. However, analysis of quantitative data provided inconclusive results. The authors argued that outcome measures, clinical stage, case severity, treatment length, and dosage, should be individually considered to better adjust the intervention to each individual’s capacity (Street et al., 2018, 2019). In a subsequent study, the authors provided further considerations on the feasibility of TIMP intervention in different home environments and the adaptability of the exercises for portable electronic devices (iPad) based on two patient cases (Street et al., 2019). Study results indicated significant improvements in motor function after 12 sessions and retention of gains particularly for the patient with less severe impairments at baseline.

Patterned Sensory Enhancement is another NMT intervention that uses rhythmic, melodic, and harmonic elements of music to provide temporal, spatial, and dynamic information about the movement (Thaut et al., 2002). In this technique, a music therapist plays a musical instrument to provide cues to facilitate the timing, force, duration, and direction of movements that are typically discrete and non-rhythmic in nature (Thaut et al., 1991; Thaut and Hoemberg, 2014). Research has shown that cueing cyclical reaching movements of the paretic arm in chronic stroke promotes a significant reduction in trajectory variability and normalizes the velocity and acceleration profiles of arm movement (Thaut et al., 2002). Recent meta-analyses and systematic reviews have indicated that rhythmically cued exercises are indeed effective to improve upper extremity function in stroke (reviewed in Ghai, 2018; le Perf et al., 2019). Clinical evidence supports the use of rhythmic cueing to enhance arm function post-stroke, with significant changes in outcome measures such as the Fugl-Meyer upper extremity assessment, Action arm reaching test, Wolf motor function test, Nine-hole peg test, Stroke impact scale, and elbow range of motion (Ghai, 2018). Optimum training dosage for upper extremity seems to involve 30 min to 1-h training sessions for a minimum of three times a week (Ghai, 2018).

Recent RCTs have provided further evidence of the effects of rhythmically cued PSE training in the recovery of upper limb function. In a recent study (Tian et al., 2020), 15 stroke patients received 30 min of training in addition to conventional therapy, while patients in the control group received 30 min of additional conventional therapy, 5 days per week, for a total of 4 weeks. The training consisted of presenting a metronome with gradually increasing frequency during the performance of gross motor function tasks, such as shoulder flexion/extension or abduction/adduction, elbow flexion/extension, arm-reaching, and grasping tasks. Post-intervention assessments indicated that patients in both groups improved from baseline, however, patients in the rhythmic cued training showed more improvement in motor function assessments including the Fugl-Meyer upper extremity assessment, the Wolf motor function test, and the Barthel Index. Moreover, surface electromyography recordings of the affected biceps and triceps revealed a significant reduction in the co-activation interval of the agonist and antagonist after cued training, particularly during elbow extension movements, which was not observed after conventional therapy. This finding suggests that rhythmically cued training helped regulate the activation pattern of agonist and antagonist muscles of the affected arm, facilitating task-oriented movements of the hemiparetic upper extremity (Tian et al., 2020). Another study examined the immediate effects of different types of auditory cueing on paretic shoulder movements after stroke (Kang et al., 2020). A group of 16 chronic stroke patients performed upper limb movements, such as shoulder abduction, holding, and adduction, in three different cueing conditions: monotonic rhythmic cues (metronome); melodic cues where changes in pitch contour reflected different movement kinematics; and no auditory cueing. Analysis of movement kinematics using inertial measurement units indicated that the musical properties of the rhythmic cues influenced the execution of paretic shoulder movements particularly during the holding phase. Overall, the findings suggested that presenting pitch contour embedded in isochronous rhythm enhanced movement positioning, decreased movement variability, and improved endurance, thus indicating that pitch information may provide additional or more salient information regarding movement spatial location (Kang et al., 2020). These findings seem to be specific to the holding phase of shoulder movement and further research is of interest to better understand how ascending and descending pitch information may affect other kinematic phases of upper limb movements.

It is also worth noting that studies examining music-based interventions for stroke often report non-motor benefits, including improved language skills (Grau-Sánchez et al., 2018), as well as increased quality of life and positive emotions (Fujioka et al., 2018; Grau-Sánchez et al., 2018; Street et al., 2020). A recent study by Haire et al. (2021b) reported significant non-motor benefits in an intervention based on TIMP for stroke patients. The study protocol involved nine sessions administered over 3 weeks to 30 stroke patients. Post-intervention assessments indicated a positive impact on affect and mood for participants in all groups, while the intervention combined with mental imagery resulted in increased mental flexibility, as measured with the Trail Making Test – part B (Haire et al., 2021b). Given the consistency of findings reporting non-motor benefits of RAS across studies, further research would be of interest to better understand the mechanisms underlying these positive cognitive effects.

#### Cerebral Palsy

Motor function and posture disorders resulting from brain damage during the development of the nervous system are common impairments that may also be accompanied by sensory, cognitive, and behavioral deficits that are predominantly addressed with rehabilitation (Boyd et al., 2001; Rosenbaum et al., 2007).

Initial clinical evidence has emerged on the effects of active musical instrument playing (i.e., piano or keyboard) to improve manual dexterity, finger, and hand motor function for persons with CP (Chong et al., 2013; Lampe et al., 2015; Alves-Pinto et al., 2016). Alves-Pinto et al. (2017) studied the effects of piano training on sensorimotor skills of a group of 16 individuals with CP (age range: 11–52 years) and a group of six typically developing youth (age range: 7–17 years). All participants received 2 h of piano lessons weekly with a piano teacher for a total of 4 weeks. Assessments using MIDI data indicated a trend toward an improvement in movement variability of keypresses for individuals with CP, however, these changes did not reach statistical significance. The authors argued that the number of sessions offered in the intervention may have not been sufficient to promote significant benefits. Another point to observe is that the primary outcome measure in this study was not a standardized and validated test to clinically evaluate hand/finger motor function, thus it may have not adequately captured possible outcome changes. More recently, Alves-Pinto et al. (2021) examined whether music instrument playing would induce changes in the brain as a result of neuroplastic processes. A single participant (16 years old) with unilateral spastic CP underwent 18 months of individualized piano training with a professional piano teacher. The post-intervention assessment revealed no significant changes in manual function as measured with the Box and Block Test and the Hand Grip assessment. Nevertheless, brain imaging examining white matter structure suggested potential changes in sensorimotor pathways after the intervention. Further research is greatly needed to better determine the best intervention protocol (dosage and duration) and the outcome measures most suitable to assess the effectiveness of active musical instrument playing as a rehabilitation tool in this population.

Recent studies have investigated the effectiveness of TIMP for motor rehabilitation of children and adolescents with CP. In Marrades-Caballero et al. (2018), 18 children and youth with severe bilateral CP aged between 4 and 16 years were randomized to standard physiotherapy treatment (control) while the second group received TIMP intervention in addition to usual care. Results suggested significant motor function improvements from baseline during the first phase of the study in measures of arm and hand positioning for participants who received TIMP while no significant change was observed for those in the control group. Further, these improvements remained stable in a follow-up assessment conducted 16 weeks after treatment was completed. Dogruoz Karatekin and Icagasioglu (2021) reported significant functional gains and improved grip strength, selective strength of the fingers, gross and fine hand motor skills after 3 months of TIMP-based piano intervention in nine adolescents with CP. These findings show promising evidence of the potential of this music-based therapy.

There is also preliminary evidence of the effects of PSE on gross motor capacity and functional strength in children with spastic CP (Peng et al., 2011; Wang et al., 2013), however recent research is limited. Peng et al. (2011) studied the immediate effects of PSE to improve muscle power and movement control during loaded sit-to-stand. It was observed that children who practiced sit-to-stands in the PSE condition presented shorter movement time, improved smoothness of center-of-mass trajectory, and increased extensor power of lower extremities compared to those practicing with no music. A subsequent randomized controlled study was developed to investigate the effects of a 6-week home-based PSE exercise program (Wang et al., 2013). Thirty-six children aged 5 to 13 with spastic diplegia were divided into two groups: a PSE group or a control group where exercises were performed without music. The study results indicated that both groups improved in measures of the Gross Motor Function Measure. However, children who exercised with PSE music presented significantly higher improvements in gross motor capacity than those who practiced without music. Moreover, gains were preserved at a 3-month follow-up (Wang et al., 2013). These findings thus indicate that auditory entrainment might act as guidance thereby improving critical gross motor skills for sit-to-stand movements. Further research would be of interest to examine the feasibility of PSE intervention for upper extremity function in this population.

#### Other Clinical Applications

Interventions involving active music playing for upper extremity rehabilitation have been extensively implemented in clinical practice (Hurt-Thaut, 2008; Altenmüller and Schlaug, 2015). However, the number of clinical trials examining the effectiveness of these techniques in fine/gross motor function is surprisingly limited and does not seem to reflect the broad use of these therapeutic techniques in the clinical context.

Fine motor disability in PD has recently gained more attention from clinicians and researchers, and preliminary findings suggest that music-based interventions may be particularly well-suited to help address these motor deficits. In a recent case study, Buard et al. (2019b) examined the effects of a TIMP-based protocol consisting of 15 sessions of bimanual exercises using a keyboard, castanets, and other percussion instruments to address fine motor skills in three PD patients. Assessments conducted pre- and post-intervention indicated significant improvements in overall motor function – as shown by a reduction in MD-UPDRS III scores – and increased fine motor skills. Additionally, neuroimaging results revealed significant increase in cortical beta-band activity and stronger functional connectivity between auditory and motor areas of the brain after 5 weeks of music-based intervention. Piano training has been implemented to address deficits in executive functions in older adults with PD (Bugos et al., 2021). Forty-five PD patients were allocated to a waitlist control group or group piano training consisting of finger dexterity exercises and learning simple piano melodies over 10 days. Cognitive assessments indicated significant improvements in the Stroop test after the training, however, no significant changes were observed in other cognitive measures such as verbal fluency (D-KEFS Verbal Fluency subtest), processing speed and cognitive flexibility (Trail Making Test – Part B, Coding and Symbol Search). No assessment of hand/finger motor function was performed as the study focused on non-motor symptoms of PD. Thus, the effectiveness and clinical guidelines for the application of active music playing for upper extremity rehabilitation in PD are yet to be determined.

Two recent studies investigated the effects of piano training on cognitive functioning in patients with TBI, however, little is yet known about its effectiveness on motor function (reviewed in Mollica et al., 2021). In Vik et al. (2018), an 8-week intervention program based on piano training was implemented for young adults (*n* = 7) with mild TBI. Although motor function was not systematically evaluated, results revealed improvements in measures of verbal learning and significant changes in brain activity in frontal regions during a music listening test (see also Vik et al., 2019). Similar results were reported in a recent RCT (Siponkoski et al., 2020), where 39 patients with moderate to severe TBI received two weekly individual sessions of 60 min duration consisting of learning to play songs on the piano and playing sequences of musical rhythms and musical exercises on the drum. Results indicated significant enhancement in executive function after 3 months of intervention, with carry-over effects at a 3-month post-intervention follow-up. In addition, brain imaging results suggested increased gray matter volume in prefrontal areas, which were correlated with cognitive improvements after the intervention (Siponkoski et al., 2020). On the other hand, no significant changes were observed in upper extremity motor functions. The authors suggested that the intervention targeted primarily cognitive function and that the study inclusion criteria focused on cognitive but not motor deficits, which may have interacted with the effectiveness of the intervention on motor-related outcomes.

The effects of piano playing for upper extremity rehabilitation in MS have been investigated in one RCT (Gatti et al., 2015). Nineteen hospitalized adults were randomly allocated into two groups: one group received 30 min of daily upper-limb rehabilitation based on keyboard music exercises for 3 weeks, while the second group received the same intervention but with the auditory feedback provided by the keyboard turned off. Post-intervention assessments of hand function indicated significant time effects for all outcome measures, however, the change in hand dexterity was significantly greater in the group who received auditory feedback than in the control group. This finding corroborates the results reported in a similar study with stroke patients (Tong et al., 2015) and highlights the key role of auditory-motor coupling to engage multisensory and motor networks during active music playing to promote neurologic recovery.

Clinical research on the application of music-based interventions specifically targeting fine and/or gross upper limb skills in elderly people is limited. Kim et al. (2017) assessed age-related changes in gross motor function with bimanual drumming tasks. Older adults with and without mild dementia performed tasks involving simultaneous or alternated bimanual movements in synchrony with a metronome. Findings revealed significant correlations between synchronization errors committed during bimanual tapping and older adults’ performance on cognitive tasks involving executive control and cognitive flexibility. Specifically, the group of adults with mild dementia presented greater synchronization errors and increased variability compared to healthy older adults and a control group of younger adults. In a subsequent study, Kim and Yoo (2020) examined the effects of a dual-task intervention for healthy older adults. Ten adults completed an 8-week intervention involving dual-task exercises such as walking or tapping in synchrony with metronome cues while playing percussion musical instruments with different rhythmic patterns or rhythmically chanting/singing. Assessments conducted pre/post-intervention evaluating gait during dual tasking showed that the intervention group exhibited decreased step length and increased step frequency after training, whereas participants who did not receive the intervention presented opposite gait patterns with increased walking speed and stride length. For the authors, this finding suggests that participants in the intervention group used a compensatory strategy to guarantee safety during cognitively demanding tasks such as walking while playing a musical instrument. Results also demonstrated a significant improvement in tasks requiring executive control of attention for participants in the intervention group, suggesting that the protocol may be effective in improving cognitive processing and gait control, which are critical to prevent falls in this population.

While research on music-based interventions for motor rehabilitation of older adults is scarce, there is growing evidence of the effectiveness of short-term musical training for cognitive rehabilitation in this population (Bugos et al., 2007; Seinfeld et al., 2013; Bugos, 2019; MacRitchie et al., 2020). Although a thorough review of the effectiveness of music-based intervention in cognitive rehabilitation is beyond the scope of this paper (for further discussion, see Hegde, 2014; Sihvonen et al., 2017; Fusar-Poli et al., 2018; Koshimori and Thaut, 2019; Schneider et al., 2019; Mollica et al., 2021), it is of note that recent intervention studies have shown significant improvements in cognitive function in healthy older adults involved in piano training programs (Bugos and Kochar, 2017; Degé and Kerkovius, 2018; Bugos, 2019; Zendel et al., 2019; MacRitchie et al., 2020; Guo et al., 2021; Worschech et al., 2021). For instance, in MacRitchie et al. (2020), 15 older adults (aged 65 years or older) participated in a piano training program consisting of ten 60-min group lessons involving learning to play simple melodies and ensemble playing tasks. Pre/post assessments included the Trail Making Test, the Jebsen Taylor hand function tests, and a visuomotor synchronization task. Results revealed significant gains in visuomotor skills as indicated by improved scores in Part A of the Trail Making Test after the 10-week intervention. However, no significant changes were observed in measures of fine motor skills. These findings indicate that a short-term intervention may be sufficient to promote positive benefits in cognitive functioning, whereas transfer of skills into general fine motor function may require longer interventions for healthy older adults (MacRitchie et al., 2020). Investigating this hypothesis is crucial to help structure standardized therapeutic protocols for specific treatment targets in this population.

## Underlying Mechanisms

Rhythm- and music-based interventions are complex multimodal rehabilitation techniques that involve multiple active therapeutic elements, varied therapeutic contexts (i.e., individual or group treatments), and treatment plans or musical exercises that are developed to target specific goals, challenges, or symptoms. Thus, all these elements can potentially contribute to the positive changes promoted by music interventions (Sihvonen et al., 2017; Altenmüller and Stewart, 2020; Brancatisano et al., 2020; Grau-Sánchez et al., 2020). The neural mechanisms underlying the motor benefits reviewed here have only started to be uncovered by experimental, neuroimaging, and neurophysiological research conducted in the past years (reviewed in Koshimori and Thaut, 2018; Damm et al., 2020). In the context of motor rehabilitation, there is converging evidence of the capacity for rhythm and music to induce neural entrainment of widely distributed auditory, sensorimotor, and motor networks of brain (Damm et al., 2020), to modulate the dopaminergic mesolimbic system (Koshimori and Thaut, 2018; Damm et al., 2020) – which is notably involved in rhythm perception, reward-based motivated learning, and affective regulation – and to promote structural and functional neuroplastic changes through active engagement with music (Altenmüller and Schlaug, 2015). In the following sections, we briefly overview current neuroimaging and neurophysiological evidence supporting the potential implications of these mechanisms on the effects of rhythm- and music-based intervention.

### Auditory-Motor Entrainment: The Role of Beta-Band Modulations

Research evidence suggests that the ability to synchronize bodily movements to external rhythmic stimuli is based on neural entrainment, whereby the repetitive firing of neurons in the brain synchronizes to the rhythm of temporally predictable events (Lakatos et al., 2019). Electrophysiological studies have shown that auditory rhythms induce entrainment in the auditory cortex as the periodicity of the neural response in the auditory system closely matches the frequency of the auditory beat (Nozaradan et al., 2011, 2012; Fujioka et al., 2012a; Nozaradan, 2014; Doelling and Poeppel, 2015; Crasta et al., 2018; Doelling et al., 2019; Bouvet et al., 2020). Moreover, the rhythmic neuronal firing remains phase-locked to the stimulus frequency even when a beat is omitted or after stimulus discontinuation, allowing individuals to predict and anticipate when the next beat will occur (Lakatos et al., 2013; Tal et al., 2017; Nobre and van Ede, 2018).

The predictability of auditory rhythms primes the motor system into a state of readiness to move and provides precise anticipatory time cues whereby movement planning and execution occurs (Thaut et al., 2015; Crasta et al., 2018). For instance, it has been shown that merely listening to auditory rhythms or music engages brain structures involved in the encoding of temporal stimuli and in movement control, such as the premotor cortex, supplementary motor area, basal ganglia, and cerebellum (Grahn and Brett, 2007; Chen et al., 2008a; Bengtsson et al., 2009; Grahn and Rowe, 2009; Fujioka et al., 2012a; Konoike et al., 2012; Merchant et al., 2015). Importantly, neuroimaging research has revealed that auditory and motor cortices are interconnected through widely distributed and hierarchically organized neural networks involving cortical, subcortical, brain stem, and cerebellar regions (Chen et al., 2006, 2008a,2008b; Schmahmann et al., 2007; Helmich et al., 2010; Fernández-Miranda et al., 2015; for reviews: Petter et al., 2016; Janzen and Thaut, 2019).

It has been proposed that the functional and anatomical connections between auditory and motor-related areas allow entrainment induced by periodic auditory stimuli to modulate the activity of a distributed network of motor and sensory structures (Buzsáki, 2009; Large et al., 2015; Thaut et al., 2015; for review, see Damm et al., 2020). Psychophysical and brain imaging investigations into rhythmic auditory-motor entrainment have shown extremely fast and temporally precise auditory projections into the motor system, entraining motor responses even below thresholds of conscious awareness and engaging complex corticocerebellar networks (Thaut et al., 1999, 2009; Roberts et al., 2000; Stephan et al., 2002; Thaut and Kenyon, 2003). A series of neurophysiological studies have shown temporally correlated modulations between auditory and motor areas, primarily in beta oscillations bands, supporting the hypothesis of coupling between auditory and motor areas through neuronal entrainment by external rhythms (Fujioka et al., 2012a,b; Ross et al., 2017; Crasta et al., 2018; Buard et al., 2019a). It has been suggested that beta-band oscillations (10–30 Hz) may indeed reflect auditory-to-motor neural coupling. In cortical sensory areas, brain oscillatory rhythms in the beta frequency range are linked to rhythm perception and reflect motor-related sensory cues (Snyder and Large, 2005; Fujioka et al., 2009, 2015; Saleh et al., 2010). Moreover, beta modulation is also associated with a range of motor behaviors (Foffani et al., 2005; Gilbertson et al., 2005; Androulidakis et al., 2007) and anticipation (Saleh et al., 2010; Jenkinson and Brown, 2011; van Ede et al., 2014; Fujioka et al., 2015; Crasta et al., 2018).

Beta modulation following rhythm- and music-based interventions have been reported in recent clinical studies (Altenmüller et al., 2009; Fujioka et al., 2012b; Buard et al., 2019b; Calabrò et al., 2019; Naro et al., 2020). A parallel-group RCT combined with electroencephalography (EEG) demonstrated that 25 participants with PD who received an 8-week treadmill gait training with RAS showed a stronger EEG power increase related to specific periods of the gait cycle and greater improvement of fronto-centroparietal/temporal connectivity in alpha and beta-bands compared to the patients who received the training without RAS (Calabrò et al., 2019). In addition, increases in the fronto-centroparietal and fronto-temporal beta connectivity were significantly correlated with improvement in functional gait assessment. The authors suggest that this extensive oscillatory recruitment may reflect the engagement of compensatory/adaptive mechanisms involving different cortical areas as well as the cerebellum that bypass or compensate deficient basal ganglia-thalamo-cortical loops in PD (discussed in Nombela et al., 2013; Koshimori and Thaut, 2018; Damm et al., 2020).

There is also evidence that external rhythms may facilitate residual activation of the basal ganglia-cortical circuitry. Studies with PD patients implanted with neurostimulators in the subthalamic nucleus (STN) further demonstrated that rhythmic auditory cues modulate the amplitude of beta oscillations of the STN during motor performance (Fischer et al., 2018; Naro et al., 2020). Naro et al. (2020) reported that patients with deep brain stimulation exhibited stronger remodulation of sensorimotor beta oscillations with gait cycle after a motor training program consisting of RAS combined with conventional physiotherapy than patients without deep brain stimulation, thus suggesting that the combination of these interventions potentiated the restoration of altered beta-band response profiles in PD.

Importantly, growing research evidence indicate that improvements in fine and gross manual skills following Music-supported Therapy (MST) are associated with modulation of beta-band frequency and stronger EEG coherence in broader cortical areas (Altenmüller et al., 2009; Fujioka et al., 2012b; Buard et al., 2019b; Ghai et al., 2021). For instance, in Altenmüller et al. (2009), 32 stroke patients who significantly improved fine and gross manual skills following 3 weeks of MST training showed more pronounced event-related desynchronization (ERD) in beta-band frequency before movement onset, which was not observed in a patient control group. Findings also showed stronger beta-band intra- and interhemispheric coherence between frontal and parietal areas compared to the control group during self-paced movements using the index finger and the whole arm (Altenmüller et al., 2009). An association between better rehabilitation outcomes and greater ERD have also been reported in recent studies (Fujioka et al., 2012b; Buard et al., 2019b; Ghai et al., 2021). In a recent case series, Ghai et al. (2021) examined neurophysiological changes induced by a 3-week intensive piano training in two participants after stroke. Using magnetoencephalography, the study reported changes in functional connectivity between the auditory and motor cortex in the affected hemisphere with increased alpha and beta-band coherence while listening passively to a trained musical piece. These neurophysiological changes were accompanied by improvements in manual dexterity (Ghai et al., 2021). However, these findings must be interpreted with caution as none of these neuroimaging studies involved a patient control group that engaged in a comparable active control intervention. Thus, further research would be of interest to better understand the mechanisms underlying changes in connectivity within auditory-motor networks induced by actively engaging in music playing.

### The Dopaminergic System: From Reward to Rhythm Perception and Production

Neuroimaging findings have suggested that the midbrain-striatal dopaminergic system may play an important role in the effects of rhythm- and music-based interventions. It is well-known that pleasant music modulates the activity in the reward-motivation brain networks and stimulates dopamine release in the striatum system (Blood and Zatorre, 2001; Salimpoor et al., 2011; Koelsch, 2014; Ferreri et al., 2019), which may explain mood-enhancing effects and improvements in quality of life after engaging in positive and rewarding experiences associated with music interventions (for review, see Sihvonen et al., 2017; Brancatisano et al., 2020; Grau-Sánchez et al., 2020; Chatterjee et al., 2021).

However, dopaminergic activity also plays an important role in rhythm perception and production (Grahn and Brett, 2007), rhythmic motor control (Miller et al., 2013; Braunlich et al., 2019; Koshimori et al., 2019), and prediction error (Friston et al., 2017; Ramakrishnana et al., 2017; Sarno et al., 2017), thus suggesting that the dopaminergic system is key to understanding auditory-motor interactions. Considering that music extensively modulates anatomical and functional connectivity between auditory areas and striatum, examining dopaminergic responses and changes in frontostriatal networks induced by music and rhythm is of interest.

Indeed, only few studies to date have investigated dopaminergic function using PET and dopamine radioligands (Salimpoor et al., 2011; Koshimori et al., 2019). In a recent study, Koshimori et al. (2019) employed [^11^C]-(+)-PHNO-PET to measure RAS-induced dopaminergic responses in the basal ganglia of eight healthy young adults during a finger-tapping task with and without RAS. Results indicated significantly greater dopamine responses in the left ventral striatum during non-RAS finger tapping compared to RAS. This result suggests that, in healthy younger adults, performing a task without auditory rhythmic cues required more motivational/attentional efforts directed toward motor timing control. This study thus demonstrated that rhythmic cues modulated dopaminergic responses in the basal ganglia, opening new avenues for further investigations into whether RAS may be able to modulate residual dopaminergic function and/or restore basal ganglia-thalamo-cortical network in PD.

### Neuroplasticity

It has been well-documented that music training and learning promote significant functional and structural changes in the brain, particularly in motor regions (Münte et al., 2002; Altenmüller and Schlaug, 2015). For instance, Pascual-Leone (2001) demonstrated that learning to play short sequences on the piano significantly changes the cortical representation of flexor and extensor finger muscles in the primary motor cortex. Considering that active music-based interventions involve motor skill acquisition, sensorimotor integration, multimodal stimulation, and extensive practice, it is hypothesized that similar activity-dependent neuroplastic changes are promoted by short periods of intervention (Altenmüller and Stewart, 2020; Grau-Sánchez et al., 2020).

There is indeed growing evidence of the direct association between neuroplasticity and functional recovery after rhythm- and music-based interventions (del Olmo et al., 2006; Amengual et al., 2013; Grau-Sánchez et al., 2013; Ripollés et al., 2016). For instance, in Ripollés et al. (2016), fMRI data of 14 chronic stroke participants indicated that improvement in fine and gross manual dexterity following MST was associated with enhanced connectivity in a network involving the precentral gyrus, supplementary motor area, inferior frontal gyrus and primary auditory cortex in the affected hemisphere as well as in the primary auditory cortex in the non-affected hemisphere when compared to healthy participants during a listening task contrasting trained/familiar music to untrained/unfamiliar music. Two experimental studies using Transcranial Magnetic Stimulation (TMS) to evaluate changes in sensorimotor representations have shown that gains in motor performance after MST training were associated with an increase in excitability of the motor system and cortical motor map reorganization in the affected hemisphere in subacute and chronic stroke patients (Amengual et al., 2013; Grau-Sánchez et al., 2013).

Emerging evidence in other clinical populations has indicated reestablishment of functional connectivity between auditory and motor regions after rhythm- and music-based intervention. Results from a neuroimaging study with 10 with neurodevelopmental disorders showed stronger endogenous connectivity from the left primary motor cortex to the right cerebellum after 18 months of piano training compared to a control patient group who did not receive any training (Alves-Pinto et al., 2015). Functional changes in cerebellar circuits were also reported in a study using PET to examine neural changes post-RAS intervention (del Olmo et al., 2006). Results demonstrated that PD patients with H&Y 1–2.5 stages exhibited an increase in the resting glucose metabolism measured by [^18^F]-FDG PET in the right cerebellum and increased activity in the right parietal (BA39) and temporal lobes compared to the brain function before the intervention. Moreover, these functional changes were accompanied with normalized finger tapping performance and gait, suggesting that RAS training strengthened corticocerebellar activity, which may be associated with compensatory/adaptive responses.

Collectively, these early findings suggest that rhythm- and music-based interventions can promote cortical reorganization and functional changes through neuroplasticity. However, these findings must be interpreted with caution as these neuroimaging studies did not involve a patient control group that engaged in a comparable active control intervention. Further research is needed to uncover the specific underlying neural mechanisms by which activity-induced plasticity is generated and to better understand whether music and rhythm modulate the residual neural resources in brain areas altered by aging or neurological disorders and/or engage unaffected brain areas to compensate for impair function. These findings will help refine the current intervention protocols and generate new hypotheses.

## Future Perspectives

Research conducted in the past decades has significantly contributed to a better understanding of how and why rhythm- and music-based interventions can be effective tools in motor rehabilitation. Nevertheless, there are several questions yet to be explored and methodological limitations that need to be addressed in future research.

Most research efforts to date have been devoted to investigating the effects of auditory rhythms on motor function in Parkinson’s disease and stroke, with findings consistently supporting the use of rhythmic auditory cues to enhance motor performance in these conditions. On the other hand, there is a paucity of high-quality randomized clinical trials with other populations, including (but not limited to) traumatic brain injury, children with cerebral palsy, Alzheimer’s disease, and older adults. The studies reviewed here provide promising behavioral evidence and lay the foundation upon which new research can be developed to better understand the effects of rhythmic auditory cueing on gait, posture, balance, and upper extremity function. Further research is critical to determine standardized protocols (cueing frequency, treatment intensity and duration, and best clinical stage) tailored to these neurological conditions. Protocol inconsistencies in relation to types and frequencies of RAS, instructions given to participants, and differences in participants’ clinical characteristics at baseline also seem to be at the root of some of the conflicting results reported.

There is increasing interest in the application of active music playing interventions to improve upper-limb functionality, manual dexterity, and fine motor skills, and growing evidence indicates that musical training is beneficial for motor rehabilitation. Research on subacute stroke has been at the forefront, as revealed by the significant number of publications in the past years, whereas the effects of music interventions to address upper-limb function in other clinical populations (e.g., traumatic brain injury, multiple sclerosis) and aging is still in the initial stages. Questions regarding the feasibility of home-based training programs, optimal training intensity and duration, best clinical stage, and the long-term sustainability of improvements should be systematically investigated. It is also important to note that many studies involving musical training (i.e., piano training) did not involve a trained music therapist to administer the intervention. Although the studies reviewed here showed beneficial effects regardless of the involvement of a dedicated music therapist, it is important to better understand the role of the therapeutic relationship between patient and therapist on the outcomes and the ethical implications of the administration of music-based treatments for individuals with neurological conditions.

In order to improve the quality of evidence, randomized controlled trials with larger samples, stratification of patients based on spared cognitive, rhythm abilities, and baseline function, as well as appropriate standardized outcomes measures are needed. One of the crucial methodological aspects that should be addressed in future research is the inclusion of active non-musical control conditions with similar motivational, mood, and emotion-inducing qualities. Particular attention should be given to ensure that experimental and control groups are similar in relation to training intensity. More information on retention of gains with long-term follow-up assessments is also necessary. Combining behavioral assessments with other objective outcome measures, including biomechanical measures (e.g., motion capture, electromyography), is key to better understanding the effects of auditory rhythms and different parameters of auditory feedback on movement kinematics and muscular activity.

Music and rhythm interventions in motor rehabilitation need to be grounded within a neurobiological understanding of the underlying mechanisms, and for that, neuroimaging studies play an important role. More neurophysiology studies are needed to determine how RAS-induced entrainment modulates brain oscillatory activity in non-auditory areas in clinical populations. Neuroimaging research is also essential to better understand the mechanisms underlying the effects of auditory rhythmic cueing on other motor-related symptoms, such as balance and freezing of gait. One hypothesis is that these beneficial effects may be partly mediated by modulation of brain activity in the pedunculopontine nucleus (PPN) (Thevathasan et al., 2012a,b; Molina et al., 2020; He et al., 2021). This subcortical region has important connections with the subthalamic nucleus, basal ganglia, and inferior colliculus, and may be able to modulate activity in dopaminergic networks (Koshimori and Thaut, 2018). Studies examining the dopaminergic function and dopamine radioligands using PET as well as with individuals undergoing neuromodulation to treat PD could shed new light on subcortical mechanisms underlying rhythm- and music-based interventions. Neuroimaging research has great potential to promote active crosstalk between basic and applied research, for instance, allowing a better understanding of how rhythm-based intervention may be extended to remediate cognitive, speech and language deficits in populations where timing and rhythm disorders are part of core symptoms of neurodevelopmental disorders, such as speech and language impairments, developmental stuttering, and autism spectrum disorder (Falk et al., 2015; Janzen and Thaut, 2018; Ladányi et al., 2020; Lense et al., 2021).

To date, brain plasticity induced by rhythm- and music-based interventions have been mostly demonstrated by comparing brain activity pre- and post-intervention. However, the recent advent of portable neuroimaging technologies that are less sensitive to motion artifacts such as Functional Near Infrared Spectroscopy (fNIRS) can provide valuable insight into brain function in freely moving participants in ecological settings (Balardin et al., 2017). Recent studies have implemented fNIRS to investigate the neural correlates associated with cued rhythmic movements in healthy young and older adults during short-term RAS training (Vitorio et al., 2018; Curzel et al., 2021). Overall, these studies demonstrated the feasibility of this neuroimaging technology to monitor brain activity during walking (Vitorio et al., 2018) and drumming to rhythmic auditory cues (Curzel et al., 2021), opening new possibilities for monitoring the relationships between neural plasticity and behavioral improvement during interventions.

Finally, another important item on the research agenda is increasing the availability of music interventions in hospitals, communities, and home settings. Recent advances in mobile technologies whereby motor behaviors can be monitored via dedicated sensors may be instrumental to implementing assistive rehabilitation strategies via apps, serious games, or touchscreen musical instruments on tablets (Dalla Bella, 2018). For instance, a recent open-label clinical trial demonstrated the beneficial effects of a 1-month (30 min/day, 5 days) gait training with auditory rhythm in a home setting with 45 patients with moderate PD via the use of a smartphone application combined with ankle-worn sensors (Cochen De Cock et al., 2021). Street et al. (2020) discussed the potential use of touchscreen devices (iPad) in acute stroke rehabilitation as tools to enhance treatment dosage and engagement. More recently, the potential benefits and challenges of adapting in-person therapeutic sessions to remote music therapy services have been examined, with recommendations for the implementation of telehealth into routine care (Cole et al., 2021). Further research to investigate how technology can be incorporated into clinical practice in music-based motor rehabilitation would be of interest.

## Conclusion

The effectiveness of rhythm- and music-based interventions in motor rehabilitation has been investigated in a growing number of studies. Converging research evidence indicates that musical rhythm is a powerful tool capable of modulating the activity of multiple brain networks and inducing neural plasticity, with great potential for supporting or recovering motor functioning. While the effect of rhythmic auditory cueing on gait performance is well-documented in PD and stroke and the clinical evidence of its effectiveness for gait training in these populations is robust, the effects of RAS on gait training in other populations (e.g., traumatic brain injury, children with cerebral palsy, and older adults) is yet to be fully examined. Similarly, Music-supported Therapy has been systematically examined in subacute stroke and there is growing evidence of its positive benefits to recover functional movement of the paretic upper extremity. Nevertheless, the understanding of the effects of active music playing for rehabilitation of fine and gross motor function in other neurological conditions is in its initial stages.

Recent neuroimaging and neurophysiological research have started the journey toward a sound neuroscientific basis for rhythm- and music-based interventions, providing a better understanding of how the brain responds to the periodicity of auditory rhythmic patterns and how movements can be shaped by rhythm. A full understanding of the mechanisms underlying the wide range of therapeutic benefits of rhythm-based musical interventions is on the research agenda for the years to come.

The body of knowledge reviewed here provides evidence of the feasibility and effectiveness of the application of rhythm and music to restore motor function in a wide variety of clinical settings. The research gaps highlighted in this article clearly demonstrate that this area of research has a large potential yet to be fully explored.

## Author Contributions

All authors listed have made a substantial, direct, and intellectual contribution to the work, and approved it for publication.

## Conflict of Interest

The authors declare that the research was conducted in the absence of any commercial or financial relationships that could be construed as a potential conflict of interest.

## Publisher’s Note

All claims expressed in this article are solely those of the authors and do not necessarily represent those of their affiliated organizations, or those of the publisher, the editors and the reviewers. Any product that may be evaluated in this article, or claim that may be made by its manufacturer, is not guaranteed or endorsed by the publisher.

## Supplementary Material

Supplementary Table 1 Summary of studies published in the past 5 years investigating the application of Rhythmic Auditory Stimulation for gait rehabilitation.

Supplementary Table 2 Summary of studies published in the past 5 years investigating the application of music-based interventions for motor rehabilitation.
