# Supplementary material for: Rhythm and Music-Based Interventions in Motor Rehabilitation: Current Evidence and Future Perspectives
Source: Front Hum Neurosci. 2022 Jan 17;15:789467. doi: 10.3389/fnhum.2021.789467 (PMC8801707; doi:10.3389/fnhum.2021.789467)
Supplement: Supplementary Table 1 — Summary of studies published in the past 5 years investigating the application of Rhythmic Auditory Stimulation for gait rehabilitation. [file Data_Sheet_1.PDF]

Supplementary Material – Table 1

Summary of studies published in the past five years investigating the application of Rhythmic Auditory Stimulation for gait rehabilitation.

Reference	Study design	RAS Intervention	Control treatment	Treatment Length	Participants (n) Clinical characteristics	Outcome measure(s)	Main results
**Parkinson's Disease**							
Bailey et al., 2018	Pre/post single group	Physical therapy + RAS (individualized metronome frequency up to +10% of preferred cadence)	N/A	5 weeks of biweekly supervised sessions (45 min duration) + 12 weeks of home-based training (30 min sessions 3 days/week). Total 17 weeks.	15 PD patients ("on" state). H & Y: 2 ± 0.5	Motor function: MDS-UPDRS III<br><br>Electromyography of lower limb muscle activity. Assessments were conducted at baseline and weeks 5 and 17.	Significant decrease in motor dysfunction as measured with MDS- UPDRS III.<br>Significant reduction in gastrocnemius lateralis bilateral asymmetry from baseline to week 17 assessment.
Calabrò et al., 2019	RCT	RAS treadmill gait training (n = 25).<br>Cueing consisted of music with superimposed salient high-pitch bell sound with a gradual increase in frequency (up to 120 bpm).	Treadmill gait training without RAS (n = 25)	30 min daily RAS sessions 5 days/week for a total of 8 weeks (in addition to conventional therapy).	50 PD patients ("on" state). H & Y: 3 ± 1	Functional Gait Assessment minimal clinically significant change.<br><br>Secondary measures included UPDRS-III, BBS, FES, 10 MWT, TUG, GQI, and EEG brain measures.<br>Assessments were performed pre/post-intervention.	RAS group showed greater improvements in measures of functional gait and motor function (UPDRS-III scores).<br>Neurophysiological results indicated stronger entrainment of beta oscillations after RAS than non-RAS training.
Capato et al., (2020a)	RCT	Multimodal balance training with RAS (n = 56).<br>Cueing consisted of metronome clicks with progressively increased frequency.	Multimodal balance training without RAS (n = 50)<br><br>General education program (n = 48)	45 min sessions conducted 2 days/week for a total of 5 weeks.	154 PD patients. H & Y stage: 1-3	Balance as measured with Mini-BESTest (MBEST)<br><br>Secondary outcomes included measures of balance and gait (BBS, TUG, TUG Dual Task, N-FOGQ, FES-I)<br>Assessments were performed pre/post-intervention and at 1-month and 6-months follow-up.	RAS-supported balance training promoted greater improvements in balance than controls, with retained gains after 6 months of intervention.<br>Greater improvements were also reported for other measures of balance (BBS, FES-I), motor function (UPDRS-III) and gait (TUG Dual-Task) compared to controls.
Capato et al., (2020b)	RCT	Multimodal balance training with RAS (n = 17).	Multimodal balance training without RAS (n = 18)	45 min sessions conducted 2 days/week for a total of 5 weeks.	35 PD patients ("on" state). H & Y stage: 4	Mini-BESTest (MBEST)<br><br>Secondary outcomes included measures of balance and gait (BBS, TUG, TUG Dual Task, N-FOGQ, FES-I)<br>Assessments were conducted pre/post-intervention and at	Both groups improved significantly from baseline, however, only the RAS-supported training showed preserved gains at 6-month follow-up.<br>RAS-training also improved other measures of balance

						1-month and 6-months follow-up.	(BBS) and motor function (UPDRS-III), whereas no changes were reported in the non-RAS group.<br>No changes were found in measures of gait function in both groups.
Chang et al., (2019)	Cross-over RCT	Stepping in place with RAS. Cueing consisted of metronome clicks set at +10% of preferred cadence.	Stepping in place without RAS	Single training session in each condition with one week washout period.	21 PD patients with/without FOG ("off" state).<br>H & Y: 2 ± 0.85	Cortical excitability assessed with paired-pulse TMS.<br><br>Stepping variability, gait velocity, cadence and stride length were assessed immediately after training.	RAS-based training had immediate effects on cortical excitability compared to control, with increase in cortical silent period and decrease in short intracortical inhibition after RAS training.<br>Greater changes were observed for patients with FOG than non-freezers.<br>Results also demonstrated immediate effects on stepping variability and gait cadence, but no significant differences were observed between training conditions.
Cochen De Cock et al., (2018)	Experimental study	Walk to a metronome and four excerpts of military marches set at +10% of preferred cadence. Patients did not receive explicit instructions to synchronize their footsteps to the music.	N/A	N/A	39 PD patients ("on" state).<br>H & Y: 2 ± 0.5<br><br>39 matched healthy controls	Clinically meaningful increase in gait velocity.<br><br>There were also measures of global cognitive functioning, rhythm abilities (BAASTA, BAT), and musicality (Gold-MSI).	Patients who exhibited stronger gait synchronization to the rhythmic cues (n = 17) had better music perception skills and were more musically trained than those who struggled to adjust their steps to the rhythmic cues (n = 22). Patients who responded well to RAS improved gait velocity and stride length, whereas patients who struggled to synchronize their footsteps showed lower gait speed and increased stride length variability.
Cochen De Cock et al., (2021)	Feasibility study	Smartphone application delivers music adapted to the patient's gait cadence with a frequency increase of up to +10% preferred cadence.	N/A	Patients walked outdoors while listening to music for 30 min, five days/week for 4 weeks.	45 PD patients.<br>H & Y: 2.4 ± 0.5	Primary assessment measures evaluated observance, safety and tolerance, usability, and enjoyment of the app.	Open-label evaluations indicated that the application is safe, easy to use, well tolerated and enjoyable.<br>Gait measurements showed significant improvements in

						Gait parameters were measured pre/post-intervention in a 6-min walk test without auditory cueing (“on” state).	gait velocity, cadence, stride length and distance from baseline.
Dalla Bella et al., (2017)	Experimental study	RAS gait training. Musical with embedded metronome set at +10% or -10% of preferred cadence. Patients did not receive explicit instructions to synchronize their footsteps to the music.	N/A	3 weekly sessions of 30 min duration conducted over 4 weeks.	14 PD patients (“on” state).<br>H & Y: 2 ± 0.7<br><br>20 matched healthy controls	Gait parameters were assessed in cued and non-cued conditions before and after the intervention and at a 1-month follow up.<br><br>Rhythmic abilities were evaluated using BAASTA and a hand tapping task.	RAS-training significantly improved measures of gait velocity and stride length in the un-cued walking condition, with carry-over effects at the follow-up assessment. Patients’ stride time variability significantly reduced after training, but the effect did not persist at follow-up.<br>Nevertheless, individual differences were observed, with patients exhibiting more severe gait symptoms impairment and poor synchronization accuracy at baseline benefiting more from RAS.
Erra et al., (2019)	Experimental study	Walk 20m in three RAS frequencies (90%, 100% and 110% of preferred cadence)	N/A	N/A	30 PD patients (“on” and “off” states)<br><br>18 matched healthy controls	Gait spatio-temporal and kinematic parameters were measured during each RAS frequency in both ON and OFF medication states.	Results indicated that RAS had immediate effects on gait spatio-temporal parameters (cadence, stride time, stride length, velocity) and phase distribution, independent from dopaminergic medication. It was also observed that motor improvements were higher at RAS set at 110% of preferred cadence, although patients with more severe impairments also benefited from RAS at 100%.
Gooßes et al., (2020)	Feasibility RCT	RAS treadmill gait training<br>(n = 15). Cueing consisted of music/metronome progressively increasing in frequency	Bike ergometer training<br>(n = 17)	20 min-session conducted over 8 consecutive days in addition to usual therapy	32 PD in-patients with DBS and without DBS<br>H & Y: 2.5	Feasibility was assessed with measures of protocol acceptance, protocol transferability into clinical routine, adverse events, and patients’ perception.	Study showed that the training protocol is feasible, safe, enjoyable, and transferable into clinical routine for both patients’ groups. No adverse events were reported.
Harrison et al., (2019)	Experimental study	5m walk with metrically accentuated music set	N/A	N/A	30 PD patients	Gait parameters were measured in three	All experimental conditions significantly influenced gait

		at 90%, 100% or 110% of preferred gait cadence.			MD-UPDRS-III: 25±10<br>(“on” state)<br><br>30 matched healthy controls	experimental conditions. In RAS condition, participants walked along a well-known folk song. In the singing condition, the music played for one verse and participants walked and sang the song after it stopped. In the third condition, participants walked while imagining the continuation of the song after it stopped.	cadence and stride length, with higher benefits at frequency above preferred cadence.<br>While walking to music set at 100% of preferred cadence increased gait variability, overt signing reduced gait variability.
Horin et al., (2020)	Experimental study	Walk with metrically accentuated music set at 100% of preferred gait cadence.	N/A	N/A	24 PD patients without FOG<br>H & Y: 2<br><br>20 PD patients with FOG<br>H & Y: 2-3<br><br>24 matched healthy controls	Gait parameters were compared in two conditions. In the RAS condition, participants walked in synchrony to the music (played uninterruptedly). In the second condition, participants listened to the entire music once and, after it stopped, they were asked to mentally sing the song and synchronize their footsteps.	Results indicated higher step time variability during RAS than mentally singing.<br>It was also reported that participants with higher gait variability at baseline benefited more from rhythmic auditory cueing, with a significant reduction in measures of gait variability independent of the cueing condition.
Lirani-Silva et al., (2019)	Longitudinal study	10m walk with and without auditory cueing. Metronome was set at 100% of baseline cadence.	N/A	N/A	25 PD patients<br>MD-UPDRS-III: 30.4±9.3<br><br>29 matched healthy controls	Gait parameters were assessed at the early disease stage. In a subset of participants (n = 9), assessments were repeated after 3 years.	Results demonstrated immediate effects of cueing on gait velocity, step length, and step time for early PD patients and healthy controls. However, RAS increased measures of gait variability.<br>A follow-up test conducted after 3 years, showed positive effects of RAS on all gait parameters, and no change in gait variability.<br>Findings thus suggest that patients with worse motor impairments benefit more from RAS.
Naro et al., (2020)	Case-controled pilot study	RAS treadmill gait training. Cueing consisted of music with progressively increasing tempo up to 120 bpm.	N/A	Training consisted of daily 30 min-session conducted 6 days/week for 4 weeks total in addition to conventional therapy.	10 PD patients with DBS<br>H & Y: 3<br><br>10 PD patients without DBS<br>H & Y: 2.5	Pre/post assessments of MD-UPDRS-III, FES, BBS, TUG, 10 MWT, and ACE-R.<br><br>In addition, EEG recordings were conducted during walking at the target frequency.	All patients improved significantly with RAS-assisted training in measures of balance, motor performance, fear of fall, and overall cognitive performance. However, patients with DBS had

							greater achievements than those without DBS in measures of gait velocity, overall motor performance, timed velocity, and balance. EEG measures showed stronger remodulation of beta oscillations in patients with DBS than without stimulation.
Park et al., (2021)	Experimental study	Walk to familiar and unfamiliar music.	N//A	N/A	20 PD patients<br>H & Y: 1-3	Gait was assessed in a 2-min walking test with familiar and unfamiliar music in two sessions.	Results indicated immediate effects of RAS on gait velocity, stride length, and arm swing amplitude irrespective of familiarity with the music. With a increase in familiarity with the stimuli from session 1 to session 2, there was a reduction in stride-to-stride variability and an increase in stride length.
Thaut et al., (2019)	RCT	Home-based RAS gait training. Cueing consisted of musical with embedded metronome clicks with progressively increasing RAS frequency.	Training discontinuation between weeks 8-16 of intervention	24 weeks of 30min daily home-based training.	60 PD patients with a history of falls.<br>H & Y: 3-4	Gait parameters were measured at baseline and weeks 8, 16, and 24 of intervention.<br>Additional measures included BBS, TUG, FES, fall index, and ankle dorsiflexion.	All patients improved in gait velocity, stride length and fall index during the initial 8 weeks. With the discontinuation of intervention for the control group, significant group differences emerged in gait spatiotemporal measures and fear of falling. However, improvements were again observed for controls once training resumed. Results indicated that RAS training significantly reduced the number of falls.
**Stroke**							
Crosby et al., (2020)	Experimental study	Walking with metronome/music set at baseline cadence frequency.	N/A	N/A	22 patients<br>(>6 months post-stroke)	Temporal gait asymmetry was assessed in a single session.<br>Rhythm perception and production abilities were measured with BAT.	Results indicated immediate improvement in gait asymmetry when walking to RAS in all patients, irrespective of rhythm abilities. However, the analysis indicated stronger effects for patients with better rhythm ability.

Gonzalez-Hoelling et al., (2021)	Historical control study	Gait training with music progressively increasing in tempo up to 110 bpm (n = 27).	Conventional physiotherapy without RAS (n = 27).	The intervention group received a combination of music-based intervention in addition to conventional therapy, with RAS gait training delivered once weekly for 60 min until hospital discharge.	55 in-patients (<21 days post-stroke)	Gait, balance, and walking ability were measured pre/post-intervention with FES, TUG, and Functional Ambulatory Category scale.	Findings revealed significant improvements in measures of gait, balance, and walking ability with no significant difference between groups at discharge. However, patients in the RAS-training group showed greater improvement in walking ability at discharge than controls.
Hutchinson et al., (2020)	Feasibility study	Music-based digital therapeutic smartphone application. Music is adapted to patients' walking ability and progressively increases the tempo.	N/A	Participants completed a single 30-min training session.	11 patients (>6 months post-stroke)	Feasibility and safety were assessed after a single session. 10MWT was also performed to examined gait parameters. A subset of participants (n = 7) completed two additional sessions.	A single training session was effective to increase gait velocity. Assessments conducted in a subset of participants who completed 3 training sessions indicated a significant increase in usual walking speed. Overall findings indicate the feasibility and safety of the application.
Lee et al., (2018)	RCT	Gait training with RAS (n = 23). Cueing consisted of sounds of different pitches applied to each leg. For the paretic side, cues were presented at 10% increase for baseline and at 5% for the non-paretic side.	Gait training without RAS in addition to conventional therapy (n = 21).	Training involved 30 min sessions conducted 5 days/week for 6 weeks, in addition to conventional therapy.	44 patients (<6 months post-stroke)	Gait parameters and balance (BBS, TUG) and lower extremity function (FMA) were measured pre/post-intervention.	Gait velocity, cadence, balance, and motor function improved significantly from baseline for both groups, however RAS training promoted greater improvements than conventional therapy. Decreases in gait asymmetry on step time were observed only in the RAS group.
Mainka et al., (2018)	RCT	RAS treadmill gait training (n = 11). Cueing consisted of music with embedded metronome with tempo slowed down from baseline.	Treadmill training without RAS (n = 13)<br><br>Bobath treatment approach (NDT) (n = 11)	Treatment consisted of 5 weekly sessions over 4 weeks with training time increasing in duration. Interventions were provided in addition to conventional therapy.	35 patients (42±23 days post-stroke)	Pre/post assessments included a 3-min walking test, fast gait speed test, and balance (IEB).	Results indicated significantly higher improvements in walking distance in the 3-min test and significantly better gait velocity and cadence in the fast gait test for RAS training than controls.
Wang et al., (2021)	RCT	Gait training with RAS (n = 30). Music or metronome was set at	Conventional therapy (n = 30)	1hr sessions were conducted 6	60 patients (≥3 months post-stroke)	Gait parameters, FMA, BBS were assessed pre/post and at week 2 of intervention.	Gait maximum velocity, cadence, stride length, FMA, and BBS improved

		the patient's baseline cadence.		days/week for four weeks.			significantly from baseline with higher improvements for RAS-training than control already at week 2 of intervention.
**Traumatic Brain Injury (TBI)**							
Sheridan et al., (2021)	Case study	Gait training with RAS. Pre-recorded music with embedded metronome was presented at predetermined frequencies (not specified).	N/A	Training consisted of 30-min sessions conducted three days/week for 3 weeks.	2 community-dwelling individuals (18 and 26 years post-TBI)	Protocol feasibility was assessed based on reports of physical fatigue, adverse events, perceived satisfaction.<br>Gait spatiotemporal parameters, balance, and mobility were assessed at baseline, post-training, and at 3-week follow-up.	Results suggest that RAS training for this population is well tolerated and with no adverse events. Changes in gait parameters varied between participants with changes observed according to therapeutic goals in measures of gait velocity, balance, and walking endurance.
Thompson et al., (2021)	Case-control feasibility study	Gait training with RAS using preferred music embedded with metronome clicks set at 5% preferred cadence.	2 weeks of usual activities	30 min sessions, 5 days/week for 2 weeks.	10 participants (1-20 years post-TBI)	Gait spatiotemporal parameters were examined at baseline, after 2 weeks (control), post-intervention, and at a 1-week follow-up. Measures included 10MWT, FGA, and Physical Activity Enjoyment Scale.	The study results that RAS training is feasible, safe, and did not present any adverse events. Outcomes suggested positive trends toward meaningful changes in functional gait, cadence, and 10 MWT time and speed, with persisted effects at follow-up.
**Multiple Sclerosis (MS)**							
Maggio et al., (2021)	Feasibility study	Treadmill training with RAS (n = 10). Music with embedded metronome progressively increased in tempo up to 120 bpm.	Conventional overground gait training without RAS (n = 10).	30 min sessions, 3 days/week, for 8 weeks.	20 persons with MS (Diagnosis: 17yrs±5)<br>EDSS: 3-6	Motor function was assessed pre/post-intervention with BBS, TUG, and 10MWT. Measures of mood and quality of life were also included.	Overall, the protocol was considered feasible, safe, and well-accepted among participants. Motor function improved significantly with RAS-assisted training, in measures of walking speed, mobility, and balance. Better mood and quality of life were also reported for patients in the RAS training.
Moumdjian et al., (2019)	Experimental study	Walking with metronome or preferred music set at different tempi ranging from 0% to +10% of preferred cadence.	N/A	N/A	28 persons with MS (Diagnosis: 17yrs±9.8)<br><br>29 healthy controls	Auditory-motor synchronization were assessed along with measures of motor function (TUG, 25FWT), gait spatiotemporal parameters and cognitive function.	Results indicated that all participants (patients and controls) were able to synchronize to the stimuli at all tempi. Patients perceived less fatigue when walking to music than to metronome, with highest synchronization

							at frequencies set from +2% to +8%. However, differences were observed in relation to severity of cognitive impairment.
Moumdjian et al., (2020)	Experimental study	Walking with metronome or preferred music set up to +10% (increments of 2%) of preferred cadence.	N/A	N/A	28 persons with MS (Diagnosis: 17 yrs±9.8)<br><br>29 healthy controls	Gait dynamics were calculated with detrended fluctuation analysis.	Results suggested that synchronization to music at higher frequencies (8% and 10% of preferred cadence) would be more adequate in rehabilitation protocols as more persistent and less rigid gait dynamic processes are engaged compared to walking to a metronome. Findings indicated that both groups (patients and controls) synchronized to music and metronome using the same underlying mechanisms.
Seebacher et al., (2017)	RCT	Rhythmic-cued motor imagery training with music (n = 34) or metronome (n = 34). RAS cueing frequency were not specified.	Treatment as usual (n = 33).	Home-based sessions of 17 min practice, 6 days/week, for 4 weeks.	101 persons with MS<br>EDSS: 1.5-4.5	Walking was assessed with T25FW and 6MWT pre/post-intervention. Secondary measures included fatigue, quality of life.	Both groups improved significantly in measures of walking speed and distance. However, improvements in measures of fatigue and quality of life were only observed in RAS-assisted training, with superior results for music-cued training.
Seebacher et al., (2019)	RCT	Rhythmic-cued motor imagery training with music and verbal cueing (n = 19) or music-cueing without verbal cues (n = 20).	Motor imagery training without cueing (n = 20).	Home-based sessions of 17 min practice, 6 days/week, for 4 weeks.	59 persons with MS<br>EDSS: 1.5-4.5	Pre/pos- intervention walking was assessed with T25FW and 6MWT.	Results indicate that motor imagery with and without RAS significantly improved walking speed and distance after training, with music+verbal cueing showing stronger effects. Fatigue and quality of life improved only for groups with RAS-assisted training compared to control.
Shahraki et al., (2017)	Pilot RCT	RAS gait training (n = 9). RAS consisted of metronome cues set at 10% of preferred cadence.	Gait training without RAS (n = 9).	Training consisted of 30-min sessions conducted 3 days/week for 3 weeks total.	18 persons with MS<br>EDSS: 3-6	Gait spatiotemporal parameters were assessed pre/post intervention.	Study findings demonstrated stronger effects of RAS on gait velocity, cadence, stride length and stride time than control.
**Cerebral Palsy (CP)**							

Kim et al., (2020)	Pilot RCT	RAS gait training with simple chords (n = 6) or complex chords (n = 7), with RAS frequency increased in 5% increments.	N/A	30-min sessions conducted 3 days/week for 4 weeks.	13 individuals with CP<br>GMFCS: levels I-II<br>Age: 20±5 years old	Gait kinematic and spatiotemporal parameters were assessed pre/post-intervention.	Findings indicated that both types of cueing significantly increased gait cadence, velocity, and stride length. Analyses of kinematic parameters showed increased extension of the hip joint at terminal stance with both RAS cues, however, improvements in range of motion of the ankle joint was greater in the complex cueing compared to simple chords.
Schweizer et al., (2020)	Experimental study	Walking with metronome set at 92.5%, 100% and 107.5% of preferred gait cadence.	N/A	N/A	24 children with CP<br>GMFCS: levels I-II<br>Age: 7-12 years old<br><br>24 matched typically developing children	Gait spatiotemporal parameters were measured during a 11m walking test while walking in synchrony with different metronome tempi.<br>Rhythm perception abilities were assessed with BAT.	No significant differences in rhythm perception were observed. Children in both groups were able to adjust their walking cadence to a faster or slower metronome frequency, regardless of their rhythm abilities. However, children with CP and lower rhythm abilities had more difficulties in trials with RAS at 92.5% and 100% of cadence.
**Alzheimer's Disease (AD)**							
Wittwer et al., (2020)	Feasibility study	Gait training with RAS. Cueing consisted of recorded music with embedded metronome with progressively changing tempo to ±10%.	N/A	45-min supervised sessions delivered at home, 2 days/week, for a total of 4 weeks.	11 community-dwelling individuals.<br>ACE III score: 66	Pre/post assessment of gait parameters during cued walking. Feasibility was also assessed.	Results indicated that the protocol was feasible, safe, enjoyable, with no adverse events. Measures of gait spatiotemporal parameters showed significant improvement in gait velocity and stride length after RAS training.
**Older Adults**							
Minino et al., (2021)	Experimental study	Walking to RAS at fixed frequencies (80bpm and 120 bpm) or frequencies adjusted in relation to preferred gait cadence (90%, 100% and 110%).	N/A	N/A	13 elderly individuals<br>Age: 65-85 years old	Gait spatiotemporal parameters and trunk displacement were measured in each cued trial.	Findings indicated that a moderate increase in RAS frequency based on each individuals' baseline cadence improved gait speed, stride length and dynamic postural stability. It was also found that fixed frequencies may not be the best cueing

							strategy for rehabilitation protocols as slow tempo tended to have detrimental effects on gait and postural stability.
Vitorio et al., (2018)	Experimental study	Walking to metronome clicks set at preferred stepping frequency.	N/A	N/A	18 elderly community-dwelling individuals<br>Age: 72±8 years old<br><br>17 young adults<br>Age: 20±1 years old	Gait parameters as well as brain activity (measured with fNIRS) were assessed during cued and uncued trials.	Gait measures demonstrated significant increase in swing time and reduced gait variability when walking with RAS in both age groups.<br>Older adults presented greater activity during cued walking in multiple motor regions of the brain compared to younger adults, however, this response reduced with exposure to the cues throughout the trials. Reduced gait variability and higher cognitive function in older adults was associated with increased brain activity during RAS walking.

ACE–R: Addenbrooke's Cognitive Examination–Revised; **BAASTA:** Battery for the Assessment of Auditory Sensorimotor and Timing Abilities; **BAT:** Beat Alignment Test; **BBS:** Berg Balance Scale; **DBS:** Deep Brain Stimulation; **EDSS:** Expanded Disability Status Scale; **EEG:** Electroencephalography**; FMA:** Fugl–Meyer Assessment; **FES:** Tinetti Falls Efficacy Scale; **FES-I:** Falls Efficacy Scale-International; **GMFCS:** Gross Motor Function Classification System; **Gold-MSI:** Goldsmiths Musical Sophistication Index; **GQI:** Gait Quality Index; **H&Y:** Hoehn and Yahr stage; **IEB:** Instrumental Evaluation of Balance; **MBEST:** Mini-BESTest; **N-FOGQ:** New Freezing of Gait Questionnaire; **UPDRS:** Unified Parkinson Disease Rating Scale; **TMS:** Transcranial Magnetic Stimulation; **TUG:** Timed Up and Go Test; **10MWT:** 10-m walking test; **25FWT:** Timed 25-Foot Walk.
