# Supplementary material for: Rhythm and Music-Based Interventions in Motor Rehabilitation: Current Evidence and Future Perspectives
Source: Front Hum Neurosci. 2022 Jan 17;15:789467. doi: 10.3389/fnhum.2021.789467 (PMC8801707; doi:10.3389/fnhum.2021.789467)
Supplement: Supplementary Table 2 — Summary of studies published in the past 5 years investigating the application of music-based interventions for motor rehabilitation. [file Data_Sheet_2.PDF]

Supplementary Material – Table 2

Summary of studies published in the past five years investigating the application of music-based interventions for motor rehabilitation.

Reference	Study design	Music-based Intervention	Control Treatment	Treatment Length	Participants (n) Clinical characteristics	Outcome measure(s)	Main results
**Stroke**							
Chong et al., 2017	Pre/post (single group)	Keyboard playing (MST)	N/A	Twelve 30-minute sessions over six weeks.	17 patients Subacute	Hand function: Jamar dynamometer, BBT, JTHFT	Significantly improved score between pre-post on the pinch subtest of the Jamar dynamometer and on the BBT.
Fujioka et al., (2018)	RCT	MST	Conventional physical training (GRASP)	1hr sessions conducted over 10 weeks (total 30 sessions).	28 patients Chronic	Motor functioning: ARAT, CMSA<br>Secondary: SIS, TMT, D-KEFS, PANAS	Minor improvements were reported in motor function in both groups.<br>Both groups improved overall quality of life and reduced negative affect. However, patients in the MST showed improvement in executive function.
Ghai et al., (2021)	Case series	MST	N/A	Three 1hr sessions per week for 3 weeks.	2 patients Chronic	Motor functioning: BBT, NHPT, JHFT, JAMAR<br>MEG	Both participants improved in NHPT, JHFT, BBT after MST training.<br>Brain measures indicated enhanced connectivity between auditory and motor areas in the affected hemisphere with MST training.
Grau-Sanchez et al., 2017	Case Study (ABAB Design)	MST	N/A	Three 1.5-hour sessions for four weeks during weeks 5-8, 13-16.	1 patient Chronic	FMA, grip strength, NHPT, BBT, ARAT, CAHAI, 3D Movement Analysis, Keyboard performance	Significant improvements in BBT, CAHAI, and finger/hand tapping velocity at the end of the first treatment period.<br>Improvements were also observed in grip strength, BBT, ARAT, CAHAI, and kinematic properties of a reaching task during the second treatment period. Some gains were maintained at three-month follow-up.
Grau-Sanchez et al., 2018	RCT	MST	Treatment as usual (OT/PT)	Twenty 30-minute sessions over four weeks.	40 patients Subacute	Motor functioning: ARAT.<br>Secondary: Fugl Myer, grip strength test, BBT, Pegboard test, CAHAI.	Both groups improved significantly in ARAT score after treatment, but not at three-month follow up.<br>Both groups improved significantly in other measures at both treatment and follow-up. No significant differences between groups.
Haire et al., 2021a	RCT Pilot study	TIMP	30 minutes active TIMP + 15 minutes motor imagery; also 30 minutes active TIMP plus 15 minutes metronome-cued motor imagery.	Nine 45-minute sessions over three weeks.	30 patients Chronic	Motor functioning: FM-UE, WMFT-FAS, MAL	All groups made significant improvement on the FM-UE; treatment group made significantly higher gains on the FM-UE than the TIMP plus metronome-cued motor imagery but not the TIMP plus motor-imagery.<br>Both the TIMP and TIMP plus motor imagery groups improved significantly on the MAL.

Haire et al., 2021b	RCT Pilot Study	TIMP	30 minutes active TIMP + 15 minutes motor imagery; also 30 minutes active TIMP plus 15 minutes metronome-cued motor imagery.	Nine 45-minute sessions over three weeks.	30 patients Chronic	Non-motor functioning: TMT A & B; SAM	All groups had significant improvements in affect & mood. Conditions with mental imagery resulted in increased mental flexibility as indicated in Trail B test results.
Kang et al., 2020	Experimental study	PSE/RAS	Movement without auditory cueing	Five trials per condition.	16 patients Chronic	Movement kinematics: Positioning, variability, endurance.	Musical auditory cueing (akin to PSE) supported positioning of movement, decreased variability, and supported movement preparation/anticipation compared to rhythmic auditory cueing alone or no cueing.
Street et al., 2018	RCT Feasibility study	TIMP	Waitlist Group	Twelve 20–30-minute sessions over six weeks.	11 patients Subacute and chronic	Motor functioning: ARAT, Nine-hole Peg Test. Also conducted qualitative interviews.	Group sizes are small for conclusive results. Adherence to treatment, retention & interview responses indicated that treatment was motivating for participants.
Street et al., 2019	Case Study	TIMP	N/A	Twelve 20–30-minute sessions over six weeks.	2 patients Subacute and chronic	ARAT, NHPT, Qualitative Interviews.	Outcomes for less impaired patients improved more than those for the more impaired patient after six weeks.
Tian et al., 2020	RCT	PSE/RAS	Conventional therapy	Twenty 30-minute sessions over four weeks.	30 patients Subacute	Motor functioning: FM-UE, WMFT, BI, sEMG	All groups saw significant improvements in motor-functioning scores; the treatment group saw greater improvement in all motor measures except the FM-UE, in which the difference approached significance ($p=0.052$). sEMG recordings indicated that the co-activation interval was decreased in the RAS group compared to the control group after treatment.
**Cerebral Palsy (CP)**							
Alves-Pinto et al., 2017	Observational	Piano lessons	Group of typically developing children who received same intervention	Eight one-hour piano lessons over four weeks.	22 Adolescents & adults	Hand-motor tests at the piano; vibration-perception tests, tests of alpha-power event-related desynchronization (ERD).	In the adolescent and adult CP groups, a significant improvement in vibration-perception occurred after the treatment period, compared to the control group. There were no significant improvements in the hand-motor piano tests or alpha ERD test.
Alves-Pinto et al., 2021	Single case study	Piano lessons	N/A	30-45 min piano lessons, twice a week, for 18 months.	1 Adolescent	BBT, Hand Grip test, keystrokes velocity Diffusor Tensor Imaging.	No significant changes in manual function were detected. Assessment of brain white matter indicated potential change in sensorimotor pathways after training.
Dogruoz Karatekin & Icagasioglu, 2021	Observational	TIMP (piano only)	Control group included typically developing matched peers.	Three months of twice-weekly 40-minute sessions.	9 Adolescents	MACS, BBT, NHPT, Jamar dynamometer, key-pressing force.	All measures showed significant improvement after treatment in the CP group.

Marrades-Caballero et al., 2018	RCT	TIMP	Standard physiotherapy/wait list	Thirteen 40-minute sessions over 16 weeks.	18 Children & adolescents	Chailey Levels of Ability	The group who received TIMP improved significantly more on the Chailey Levels of Ability test than the standard treatment group both after the treatment was completed, and at 16-week follow-up.
**Parkinson's Disease (PD)**							
Buard et al., (2019)	Case study	TIMP, RAS	N/A	Fifteen sessions administered three times/week over five weeks.	3 PD patients ("on" stage) H & Y: 2-3	UPDRS III, GPT, finger-thumb opposition. MEG	Fine motor function improved significantly after TIMP. Brain imaging during cued finger tapping showed significant changes in cortical beta band activity and increased functional connectivity between the auditory and motor cortices.
Bugos et al., 2021	RCT	Piano Training	Waitlist	Thirty hours of piano/music theory training over 10 days.	45 PD patients H & Y: 1-2	Battery of cognitive tests including TMT, D-KEFS test.	Those who completed the piano training demonstrated enhanced performance on Stroop and musical self-efficacy measures.
**Traumatic Brain Injury (TBI)**							
Siponkoski et al., 2020	Crossover RCT	TIMP, MST	Waitlist	Twenty 60-minute sessions over 10 weeks.	39 Moderate & severe TBI, minimum two years post injury	NOS-TBI, GOSE, FAB, NLT, Auditory N-Back Test, Simon Task, SART, WMT-II, Digit Span test, BBT, ARAT, PPTB, structural MRI	FAB score increased significantly in the treatment group and improvements were maintained at 6-month follow up. This was correlated with increases in gray matter volume in the inferior frontal gyrus during the intervention (vs. control) period for both groups.
Vik et al., 2018	Within & between-group comparison study	MST	Healthy comparison groups	Sixteen 30-minute piano lessons over eight weeks.	7 Mild TBI, minimum two years post injury	Semi-structured interviews; MMS, CVLT 2, Stroop test, fMRI	Treatment group showed improvement in self-reported emotional and social functioning after treatment. There were also significant improvements in CVLT 2 scores, and several brain areas as seen in fMRI (medial orbitofrontal gyrus, middle frontal gyrus, anterior insular cortex, anterior and posterior medial orbitofrontal gyrus, supplementary motor area, and rostral anterior cingulate cortex).
**Older Adults**							
Bugos and Kochar, 2017	Pilot study	Group piano training	N/A	3hr-sessions conducted over 2 weeks, totaling 30hrs of training.	34 older adults	Cognitive function: WASI-IV, D-KEFS, RAVLT	Short-term training promoted significant improvements in verbal fluency and processing speed.
Bugos, 2019	Observational	Active music playing involving piano instruction,	N/A	Participants had up to 3hr of training weekly over 16 weeks.	135 healthy older adults	Cognitive function: WASI, TMT, PASAT, D-KEFS, Stroop	Significant improvements were reported for participants in the piano and percussion training in measures of bimanual synchronization and working memory. Piano training promoted higher gains in

		percussion instruction or music listening.				Motor function: finger tapper test, bimanual coordination task	motor synchronization skills than the other music-based interventions.
Degé and Kerkovius, 2018	RCT	Group drumming program	Literature program or no training	1h-session conducted once a week for 15 weeks	24 healthy older adults	Cognitive function: WASI-III, digit span backward, working memory scale, verbal memory list-learning	Music training had a positive effect on verbal and visual memory in relation to the control interventions, however, no effects were observed in measures of working memory.
Guo et al., 2021	RCT	Keyboard harmonica (i.e., melodica) instruction	Treatment as usual group	Sixteen 1-hour group lessons for sixteen weeks plus individual practice.	66 healthy older adults	MMSE, LM, digit span, VFT, TMT, PEG, WHO-5, K6, social lifestyle questionnaire, outing frequency questionnaire, fMRI	Intervention group improved significantly more on verbal recall tests. Intervention group also saw changes in functional connectivity (FC) and brain activation in several areas (FC, indicating enhanced neural efficiency after the intervention.
Kim et al., (2017)	Experimental study	Bimanual drum tapping	N/A	N/A	20 healthy older adults<br><br>17 older adults with mild dementia<br><br>21 young adults	Inter-tap intervals variability and synchronization errors.<br>Cognitive function: TMT, digit span forward	Cognitive decline was associated with higher synchronization errors. Higher variability during bimanual tapping requiring alternation of hands at slow cueing tempo was associated with more errors on cognitive tasks of executive control and cognitive flexibility.
Kim and Yoo (2020)	RCT pilot study	Dual task training with RAS	Did not receive intervention	30-min sessions administered twice a week for 8 weeks.	20 healthy older adults	Cognitive function: TMT, WCST<br>Motor function: TUG, ABC, Walking tasks	Gait ratios were significantly different between groups during a dual task. Significant improvements in TMT-B were reported for the intervention group.
MacRitchie et al., 2020	Experimental study	Group piano instruction	Wait list	One hour of weekly training plus at-home practicing for ten weeks.	17 healthy older adults	M-ACE, DASH, TMT, JTHFT, visuomotor synchronization task, semi-structured interviews	There were significant improvements on the TMT-A, though declines on the TMT-B after the training period. Qualitative data indicated that motivation was highest when songs were familiar and when participants liked their group members.
Worschech et al., 2021	RCT	Piano playing	Music listening/Music culture groups	One hour of weekly training for six months in dyads, plus ~30 day of individual practice.	156 healthy older adults	International Matrix Test	Both groups saw improvements in binaural speech in noise conditions. The speech reception threshold decreased in the left ear for the piano group.
Zendel et al., 2019	Experimental study	Piano training	Video game training & no-contact control group.	At least 30 minutes, 5 days per week, over 6 months of Synthesia (online piano training program)	34 Older adults	Speech-in-noise task with EEG	The piano training group improved significantly on their ability to hear speech in loud noise from pre- to post-training, with correlates in increased positivity in frontal-lobe electrodes for the music group.

ABC: Activities-Specific Balance Confidence; **ARAT**: Action Research Arm Test; **BBT**: Box & Block Test; **BI:** Barthel Index for Activities of Daily Life; **CAHAI**: Chedoke Arm and Hand Inventory; **CMSA:** Chedoke–McMaster Stroke Assessment; **CVLT 2:** California Verbal Learning Test; **DASH:** Disabilities of Hand, Shoulder, & Arm Questionnaire; **D-KEFS:** Delis–Kaplan Executive Function System battery; **FAB:** Frontal Assessment Battery; **FM-UE**: Fugl-Myer – Upper Extremity; **GOSE:** Glasgow Outcome Scale Extended; **GPT:** Grooved Pegboard Test; **GRASP:** Graded Repetitive Arm

Supplementary Program; **H&Y:** Hoehn and Yahr stage**; JTHFT**: Jebsen-Taylor Hand Function Test; **K6:** Kessler Measure of Psychological Distress; **LM:** Logical Memory; **M-ACE:** Addenbrooke's Cognitive Examination III Mini Version; **MACS:** Manual Ability Classification System; **MAL:** Motor Activity Log; **MEG:** Magnetoencephalography; **MMS:** Mini Mental Status Test; **MMSE:** Mini-Mental State Examination; **NHPT**: Nine Hole Pegboard Test; **NOS-TBI:** Neurological Outcome Scale for Traumatic Brain Injury; **OT:** Occupational Therapy; **PANAS:** Positive and Negative Affect Schedule; **PASAT:** Paced Auditory Serial Addition Task; **PASE:** Physical Activity Scale for the Elderly; **PEG:** Pegboard Test; **PT:** Physiotherapy; **SAM:** Self-assessment Mannequin; **RAVLT:** Rey Auditory Verbal Learning Test; **SART:** Sustained Attention to Response Task; **sEMG***:* Surface electro-myography recordings; **SIS:** Stroke Impact Scale; **TMT:** Trail-Making Test; **TUG:** Timed Up and Go; **UPDRS:** Unified Parkinson Disease Rating Scale; **VFT:** Verbal Functioning Test; **WASI:** Wechsler Abbreviated Scale of Intelligence; **WCST:** Wisconsin Card Sorting Test; **WHO-5:** World Health Organization Well Being Test; **WMFT-FAS:** Wolf Motor-Function Test – Functional Ability Scale; **WMS-III:** Wechsler Memory Scale III.
